# Synergies and Trade-Offs in Modern Poultry Nutrition: An Integrated Framework Linking Animal Health, Productivity and Greenhouse Gas Mitigation

**DOI:** 10.3390/vetsci13080789

**Published:** 2026-08-07

**Authors:** Dexin Zhao, Haoliang Chai, Linfeng Zhou, Ruicheng Han, Weiqi Peng, Hongzhi Wu

**Affiliations:** 1 Tropical Crops Genetic Resources Institute, Chinese Academy of Tropical Agricultural Sciences, Haikou 571101, China; 2 College of Animal Science and Technology, Northeast Agricultural University, Harbin 150030, China

**Keywords:** poultry nutrition, nutrient retention, precision feeding, life cycle assessment, greenhouse gas mitigation, sustainable production

## Abstract

Poultry nutrition simultaneously affects animal health, productive efficiency, product quality and the environmental burdens associated with feed production and manure management. Nutritional interventions may generate synergies among these outcomes, but they can also shift costs or environmental impacts across biological and supply-chain boundaries. This review critically evaluates low-protein diets, functional additives, feed enzymes, alternative protein sources, precision-feeding technologies and nutritional approaches to manure-emission mitigation. The evidence indicates that improved intestinal function and nutrient retention can reduce feed requirements and nutrient excretion, whereas excessive protein restriction, unstable additive responses, limited alternative-ingredient supply and poorly validated digital systems may compromise performance, product quality or economic feasibility. Environmental benefits are also sensitive to ingredient origin, processing, transport, crystalline amino acid and additive manufacture, coproduct allocation and life cycle assessment assumptions. To integrate these considerations, we propose a four-level conceptual decision framework comprising physiological adequacy, precision nutrient supply, constraint-specific functional intervention and whole-chain verification. The framework treats health, productive performance, product quality, economic feasibility and environmental impact as simultaneous decision criteria, while genotype, production stage, disease pressure, climate, ingredient availability and market objectives define the conditions of application. Standardized life cycle assessment and commercial validation are required to distinguish reproducible net benefits from improvements confined to individual indicators. This approach provides a structured basis for selecting poultry nutritional strategies that remain biologically effective, economically accessible and environmentally beneficial under practical production conditions.

## Simple Summary

Poultry nutrition influences flock health, productive efficiency and the environmental footprint of meat and egg production. These outcomes are closely connected but do not always improve together. For example, moderate dietary protein reduction can decrease nitrogen excretion, whereas excessive restriction may impair growth and product quality. Functional additives may support intestinal health and feed efficiency, but their effects vary with product composition, dose, feed processing and farm conditions. Alternative protein sources may reduce reliance on conventional ingredients, although limited supply, processing costs and inconsistent nutritional value can restrict their commercial use. This review proposes a four-level decision framework for evaluating nutritional strategies. Diets should meet physiological and productive requirements, provide balanced digestible nutrients, use functional interventions only for defined constraints, and undergo whole-chain environmental and economic verification. Life cycle assessment is used to determine whether on-farm benefits are retained across ingredient production, additive manufacture, feed processing, transportation and manure management. The framework emphasizes that nutritional strategies should be selected according to reproducible whole-system benefits rather than improvements in a single health, production or environmental indicator.

## 1. Introduction

Poultry meat and eggs provide widely accessible, high-quality animal protein with generally lower land use and greenhouse gas (GHG) emission intensity than products from ruminant systems [1,2,3,4]. Continued growth in global demand, however, means that even a comparatively efficient sector exerts substantial cumulative pressure through feed-crop production, land-use change, energy use, manure management and nutrient losses [5,6,7]. At the same time, restrictions on antibiotic growth promoters, volatile feed costs and increasingly stringent environmental expectations require poultry production to maintain flock health and productivity while reducing its resource and emission burden [8,9,10]. These objectives are often discussed separately, although they are biologically and economically interdependent [9,10,11]. Nutrition is a plausible point of integration because it simultaneously shapes intestinal function, nutrient retention, productive performance and the quantity and composition of excreta [12,13,14,15]. Improved intestinal barrier integrity and microbial stability can reduce inflammatory maintenance costs and increase the proportion of dietary nutrients retained in meat or eggs [16,17]. Better nutrient retention lowers feed use per unit of product and reduces the nitrogen and phosphorus available for ammonia, nitrous oxide and downstream eutrophication [16,17,18]. Precision balancing of digestible amino acids, energy and minerals may therefore generate synergies among health, productivity and environmental performance [19,20,21].

Such synergies are not automatic. Nutritional strategies frequently redistribute costs or environmental burdens rather than eliminating them [22,23]. Excessive crude-protein reduction may decrease nitrogen excretion but impair growth, breast yield, intestinal integrity or meat quality when essential and non-essential amino acid supply is inadequate [24,25,26]. Crystalline amino acids facilitate protein reduction, yet their fermentation, separation and drying create upstream energy and GHG burdens [27,28]. Alternative proteins may reduce reliance on imported soybean meal but can be limited by inconsistent supply, processing requirements, lower digestible nutrient density or higher cost [29,30,31]. Functional additives may improve health and feed efficiency, although variable composition and context-dependent responses weaken the certainty of their environmental return [32]. These examples illustrate a trade-off as an improvement in one objective that compromises another objective or transfers a burden to a different life cycle stage [33]. GHG mitigation warrants particular attention because feed production commonly represents the dominant share of the cradle-to-farm climate impact of poultry products, while manure nitrogen contributes further direct and indirect emissions [34]. Nutrition drives environmental mitigation via three distinct mechanistic routes. Boosted feed efficiency lessens the volume of agricultural crops, processing power and transportation inputs consumed to generate each unit of edible livestock output [35]. Amino acid-balanced diets can reduce nitrogen excretion and the substrates available for NH_3_ volatilisation and N_2_O formation, whereas improved phosphorus availability can reduce phosphorus losses and eutrophication potential [36]. Strategic raw material procurement paired with circular recycling of safe by-products from agricultural manufacturing eases pressures tied to land occupation and waste disposal [37]. Overall environmental gains shift drastically based on production context, requiring holistic evaluation that accounts for feed raw material provenance, feed manufacturing workflows, additive production processes, animal growth metrics and manure handling protocols, rather than narrow assessment centred solely on individual farm-scale metrics.

In this review, the proposed integrated framework is a conceptual decision model, not a new experimental method and not merely the organizational structure of the manuscript. It connects four sequential levels of nutritional decision-making: safeguarding minimum physiological and productive requirements; improving the precision of digestible nutrient supply; applying functional interventions to defined biological constraints; and verifying whole-chain environmental effects through life cycle assessment (LCA) [37]. Health, productivity and GHG mitigation are treated as simultaneous decision criteria, while economic feasibility and commercial scalability define whether an intervention is implementable. This differs from conventional narrative reviews that catalogue technologies within isolated domains, because the framework is designed to identify shared mechanisms, reveal burden shifting and define the conditions under which an apparent benefit remains a net system-level gain.

Important evidence gaps currently restrict this integration. Many studies assess growth, intestinal health, or emissions independently, use inconsistent functional units and system boundaries, and rarely include the upstream impacts of feed additives or crystalline amino acids [38,39]. Short, controlled trials may also overestimate responses that are less reproducible across genotypes, production stages, feed forms and commercial environments [39]. Economic analyses, pellet-quality measurements and uncertainty estimates are frequently absent [40]. Consequently, the literature supports numerous promising interventions but provides less certainty about their comparative value, application boundaries and combined effects under commercially realistic conditions.

Accordingly, this review aims to (i) critically evaluate nutritional strategies with the potential to improve poultry health, productive efficiency and environmental sustainability simultaneously; (ii) identify synergies, conflicts, boundary conditions and sources of uncertainty among these objectives; and (iii) present an integrated conceptual framework that organizes nutritional decisions from physiological adequacy to whole-chain LCA verification. By linking biological mechanisms with commercial feasibility and environmental accounting, the review seeks to support selection of interventions that provide reproducible net benefits rather than isolated improvements in individual indicators.

## 2. Health Improvement: Nutritional Modulation as a Tool for Disease Prevention and Immune Enhancement

### 2.1. Intestinal Health: Primary Target of Nutritional Regulation

Global prohibition of AGPs, coupled with growing public health threats posed by antimicrobial resistance (AMR), has revolutionized health management paradigms for intensive poultry production [41]. Modern flock health management has shifted beyond simple elimination of overt clinical diseases toward long-term stabilization of intestinal architecture and systemic physiological homeostasis [42,43]. Within this evolving industrial and public health landscape, intestinal health has emerged as the central intervention target for nutritional modulation, antibiotic-free rearing and immune homeostasis maintenance in commercial poultry systems [44,45]. Functionally dual-purpose, the poultry intestine serves as the primary site for nutrient digestion and energy turnover while housing the body’s largest and most immunologically active mucosal tissue [46,47]. Mucosal structural integrity and functional stability therefore fundamentally determine flock growth performance, stress resilience and disease susceptibility under intensive rearing conditions [48,49].

Far from a passive microbial community inhabiting the gut lumen, intestinal microbiota functions as a dynamic host-interactive metabolic entity that co-regulates avian physiological homeostasis throughout the production cycle [50,51,52]. Microbial metabolism continuously generates a spectrum of bioactive metabolites, including short-chain fatty acids, bile acid derivatives, functional amino acid metabolites, vitamins and polyamines, which extensively mediate intestinal barrier reconstruction, oxidative stress mitigation, energy metabolism balance and immune cell activation [53,54]. A structurally balanced microbial community sustains tight junction stability, restrains excessive intestinal inflammation and preserves mucosal immune tolerance across developmental stages. In contrast, microbial dysbiosis, featured by depleted beneficial taxa and overproliferated opportunistic pathogens, triggers a cascade of adverse physiological consequences [55,56,57]. Impaired nutrient digestibility, compromised feed conversion efficiency, and aggravated stress-induced intestinal damage commonly occur under dysbiotic conditions, markedly increasing susceptibility to bacterial infections [58,59]. Critically, dysbiosis-disrupted nitrogen metabolism accelerates the accumulation of intestinal toxic nitrogen compounds and elevates fecal nitrogen excretion, inevitably increasing GHG emissions from poultry farming and forming a self-reinforcing vicious cycle linking intestinal dysfunction, productivity loss and environmental degradation [60,61].

The gut–liver axis constitutes a core signalling cascade bridging intestinal microecological metabolism, systemic immune activation and inflammatory transduction in poultry [62,63]. Intestinal metabolites, microbial derivatives and luminal endotoxins translocate to the liver via portal venous circulation, exerting sustained regulatory effects on hepatic inflammatory status, lipid metabolism and whole-body nitrogen homeostasis [64,65]. Intestinal dysbiosis-induced endotoxemia persistently activates hepatic pro-inflammatory signalling pathways, driving chronic low-grade systemic inflammation. This subclinical pathological state profoundly constrains genetic growth potential, impairs systemic immune competence, exacerbates invalid nutrient consumption and increases breeding-related pollutant emissions [66,67,68]. Nutritional strategies that stabilize intestinal microecology, repair mucosal barrier damage and harmonize gut–liver metabolic crosstalk therefore provide robust foundational support for healthy, sustainable and antibiotic-free poultry production.

### 2.2. Probiotics, Prebiotics and Synbiotics

Microecological nutritional modulation underpins the technical framework of modern antibiotic substitution systems, with probiotics, prebiotics and synbiotics recognized as the most well-validated and industrially viable functional additives in poultry production [69,70]. These biological regulators restore intestinal microecological equilibrium, reinforce mucosal barrier integrity and strengthen immune competence through distinct yet complementary microbial modulation mechanisms [69]. Despite well-documented health-promoting properties, field-scale applications exhibit pronounced strain specificity, context-dependent efficacy and variable environmental adaptability under commercial rearing conditions, necessitating rigorous empirical evaluation and rational assessment of practical application trade-offs.

Probiotics are live microorganisms that confer a health benefit when administered in adequate amounts; however, their viability during feed manufacture, storage and gastrointestinal transit is strain- and formulation-dependent [71,72]. In commercial poultry farming, *Bacillus* and *Lactobacillus* represent the most adaptable and industrially valuable probiotic genera [73,74]. Spore-forming *Bacillus* strains produce stress-resistant endospores capable of tolerating high-temperature feed pelleting, gastric acid erosion and bile salt stimulation, maintaining stable viability throughout feed processing and gastrointestinal transit [75]. As a representative functional strain, *Bacillus amyloliquefaciens* enhances intestinal antioxidant capacity via Keap1/Nrf2 signalling cascade activation, effectively alleviating oxidative mucosal damage induced by intensive rearing stress [75,76]. By comparison, Lactobacillus plantarum optimizes jejunal morphological structure, improves nutrient digestibility, enhances humoral immune responses and stabilizes whole-flock feed conversion performance under conventional commercial production scenarios [77,78].

Prebiotics are indigestible feed ingredients that reach the hindgut intact, selectively facilitating the proliferation of indigenous beneficial bacteria while inhibiting the adhesion and colonization of opportunistic pathogens [79,80]. Oligosaccharides, including mannan oligosaccharides and fructo-oligosaccharides, are the most empirically validated prebiotic resources for poultry application [81,82]. These functional carbohydrates enrich intestinal beneficial genera such as Lactobacillus and Bifidobacterium, promote endogenous short-chain fatty acid biosynthesis, and thereby strengthen mucosal barrier integrity and immune homeostasis [81]. Synbiotics are compound formulations integrating screened probiotic strains and matched prebiotic substrates [83]. Prebiotics act as exclusive carbon sources for targeted probiotic proliferation, theoretically generating synergistic effects on microbial community stabilization and intestinal functional improvement, although their field efficacy remains highly dependent on rearing conditions and formula compatibility [83,84].

Probiotic efficacy displays strong strain specificity, with intraspecific functional variations often outweighing generic genus-level traits, which frequently leads to overgeneralized interpretation of experimental data [85,86]. Commercial intensive environments, characterized by persistent stress interference, complex dietary backgrounds and high ambient pathogen loads, substantially hinder stable probiotic colonization, making ideal laboratory outcomes difficult to replicate under practical farming conditions [87,88]. Dose–response relationships also remain poorly defined in industrial feeding systems [89]. Low supplementation levels fail to produce measurable physiological benefits, whereas excessive probiotic intervention disrupts indigenous microbial competitive balance and destabilizes intestinal microecological homeostasis, with variable tolerance across poultry breeds and growth stages [89,90].

### 2.3. Plant-Derived Additives (Phytochemicals)

Driven by global AGPs prohibition, plant-derived additives have emerged as a core research hotspot and industrial alternative for antibiotic substitution in green poultry farming [91,92]. Their low residual characteristics, multi-target regulatory properties and superior biological safety support broad application prospects within sustainable livestock systems [92,93]. This functional category encompasses diverse bioactive substances, including plant polyphenols, flavonoids, terpenoids, essential oils and compound herbal extracts [91,93]. These phytochemicals coordinate antioxidant, anti-inflammatory, antibacterial and digestive-promoting functions to comprehensively optimize intestinal health and systemic immune competence, evolving into indispensable functional nutrients for high-quality and eco-friendly poultry production [91,93,94].

Multiple synergistic signalling pathways underpin the physiological benefits of plant-derived additives in poultry nutrition. Efficient free radical scavenging combined with Nrf2/HO-1 cascade activation enhances intestinal antioxidant defence, effectively mitigating oxidative mucosal damage induced by intensive rearing stress [95,96]. Suppressed NF-κB pathway activation and subsequent downregulation of pro-inflammatory cytokines alleviate persistent low-grade intestinal inflammation, sustaining long-term immune homeostasis [96,97]. These phytochemicals also constrain pathogenic proliferation by disrupting bacterial membrane integrity and interfering with intracellular metabolic processes [97]. Additionally, they stimulate endogenous digestive enzyme secretion, substantially improving dietary nutrient digestibility and utilization efficiency [95]. Empirical studies have validated the efficacy of typical plant-derived components across commercial scenarios. Polyphenol complexes from grape pomace, olive pomace and tea reshape dysbiotic gut microbiota, enhance immune resilience and reduce tissue oxidative stress [98,99,100,101]. Quercetin modulates lipid metabolism and suppresses excessive abdominal fat deposition [102,103]. Rutin repairs damaged intestinal villus structure, strengthens mucosal barrier function and activates antioxidant signalling [104,105]. Nanoencapsulated rosemary essential oils overcome the low bioavailability limitations of conventional plant extracts, simultaneously improving body weight gain, protein digestibility and carcass quality while reducing abdominal fat accumulation [106,107].

The commercial application of plant-derived additives is constrained primarily by product standardization and delivery rather than by an adverse property shared across the entire phytochemical class. Concentrations and ratios of active constituents vary with plant genotype, geographical origin, harvest stage and extraction procedure, making results obtained with one preparation difficult to extrapolate to another. Stability during feed manufacture and storage also differs among compounds because oxidation, volatilisation and thermal degradation can alter the dose that reaches the animal. Limited water solubility, binding to dietary proteins or fibre, and interactions with other feed components may further reduce intestinal availability [108]. The biological response therefore depends on the chemically characterized formulation, processing stability, dietary matrix and release of active constituents at the intended gastrointestinal site. Encapsulation or other delivery technologies may improve stability and site-specific release, but their effects remain product- and process-dependent [106,107]. Recommendations should consequently be based on stability-adjusted inclusion levels and experimentally defined effective dose ranges for the specific formulation and production conditions. Adverse responses observed at excessive inclusion levels reflect the general principle that nutritional interventions have finite effective ranges rather than a limitation unique to phytochemicals [109,110].

### 2.4. Organic Acids

Featuring stable chemical properties, cost effectiveness and wide environmental adaptability, organic acids serve as fundamental intestinal functional regulators, particularly suitable for young poultry with immature intestinal structures and fragile physiological functions [111,112,113,114,115,116]. Multidimensional comparison of the efficacy of different organic acid products is shown in Table 1. Common commercial formulations, including formic acid, propionic acid, citric acid and butyric acid, exert complementary regulatory functions across different gastrointestinal segments [117,118,119]. Formic and propionic acid reduce upper gastrointestinal pH, inhibit the proliferation of Gram-negative pathogens, and lower intestinal infection risks [117]. Citric acid enhances the solubility and bioavailability of mineral elements such as calcium, phosphorus and zinc, alleviating prevalent mineral deficiency in intensive feeding systems [118,119]. Distinct from other organic acid subtypes, butyric acid acts as the preferential energy substrate for intestinal epithelial cells [118]. It promotes villus proliferation and structural repair, upregulates tight junction protein expression, reduces mucosal permeability, and blocks systemic endotoxin translocation, occupying an irreplaceable position in maintaining gut–liver axis homeostasis and repairing damaged intestinal mucosa [120].

The efficacy of organic acid products depends on acid identity, pKa, chemical form, inclusion level and release profile rather than on total acid concentration alone. Free acids and their salts differ in dissociation behaviour, palatability and activity across gastrointestinal compartments, whereas buffering or encapsulation can delay release and extend acidification effects to more distal intestinal regions [121,122]. Feed-processing temperature, storage conditions and volatility may alter product recovery, and the acid-binding capacity of the basal diet can partly neutralize the expected reduction in gastrointestinal pH. Interactions with dietary minerals, enzymes and other additives may further modify antimicrobial activity and nutrient availability. Commercial recommendations should therefore specify the acid composition and chemical form, processing stability, target release site, dietary buffering context and experimentally validated effective dose range for the intended age and production conditions. Excessive acidification can impair palatability or digestive function [123], but this represents a general consequence of exceeding an effective dose range rather than an inherent disadvantage unique to organic acids. The more relevant limitation is a mismatch among product chemistry, dietary matrix, release site and the biological constraint being targeted.

### 2.5. Immunonutrition: Functional Amino Acids and Micronutrients

Traditional nutritional frameworks categorize amino acids, vitamins and trace minerals merely as basic nutrients supporting growth and development [124]. Modern poultry immunonutrition research, however, reveals that intensive rearing stress, recurrent pathogen exposure and high-density housing fundamentally reshape host metabolic priorities [125]. Immune cell proliferation, inflammatory activation and sustained antioxidant consumption markedly elevate nutritional requirements under commercial production conditions, exceeding the supply capacity of conventional empirical dietary formulas [126,127]. Immunonutrition focuses on targeted supplementation of specific functional nutrients to remodel immune metabolism and enhance disease resistance under stress or subclinical pathological states [126].

Functional amino acids constitute the core components of poultry immunonutrition, with arginine, glutamine and threonine dominating key immune-regulatory functions. Glutamine serves as the primary energy substrate for intestinal epithelial cells and peripheral lymphocytes [128]. Endogenous glutamine synthesis fails to meet metabolic demands under intensive stress, making exogenous supplementation indispensable for sustaining mucosal immune activity and intestinal repair capacity [129]. Arginine participates in nitric oxide synthesis, mediating macrophage bactericidal activity and host anti-infective immunity [130]. Threonine acts as a critical precursor for intestinal mucin synthesis, maintaining mucus layer integrity and constructing the frontline physical barrier against pathogen adhesion and invasion [131].

Vitamins and trace minerals collaboratively construct the systemic antioxidant and immune regulatory network essential for poultry health maintenance. Vitamin A preserves mucosal epithelial structural integrity, while vitamin C and vitamin E synergistically constitute the endogenous antioxidant system to eliminate stress-induced reactive oxygen species [132,133]. Selenium, zinc and copper function as essential cofactors for antioxidant enzymes, participating in immune cell proliferation and functional activation [134]. Deficiencies of these micronutrients inevitably trigger immunosuppression and increase disease susceptibility in intensive flocks. Micronutrient regulation follows strict dose-dependent rules. Nutrient deficiency induces immune dysfunction, while over-supplementation causes oxidative toxicity and antagonistic mineral interactions. This narrow effective dosage range represents a pivotal trade-off that must be fully addressed in precision nutritional modulation.

### 2.6. Translational Stability and Commercial Validation of Nutritional Interventions

Evidence for probiotics, phytochemicals and organic acids is generally strongest for specific products tested under defined dietary, environmental or disease-challenge conditions rather than for additive classes as a whole. Commercial efficacy depends on the biologically available dose delivered to the target site rather than solely on the formulated inclusion rate. For probiotics, the relevant exposure is the viable count retained after feed manufacture, storage and gastrointestinal transit; for organic acids, essential oils and phytochemicals, release kinetics, volatility, oxidation and dietary matrix binding influence effective exposure. The responses to functional amino acids and micronutrients are similarly affected by nutrient bioavailability, dietary interactions and the physiological status of the flock. Product identity, active-component standardization, basal diet, pelleting conditions, storage duration, environmental stress and pathogen pressure therefore contribute to the heterogeneity observed among studies. Non-linear dose–response relationships are also common: subthreshold inclusion may be ineffective, whereas excessive supplementation can reduce feed intake, disturb microbial or nutrient balance, or impose unnecessary costs.

Industrial recommendations should consequently be supported by clearly defined product and batch specifications, post-processing recovery, end-of-shelf-life stability and dose-ranging studies that include a commercially realistic reference level. Multi-site evaluations should represent contrasting thermal and health conditions, use the pen as the experimental unit, and report productive performance, health status, mortality, medication use, pellet quality and economic cost. Recommended inclusion levels should be expressed as stability-adjusted dose ranges with clearly defined conditions of use rather than as universal optima. Evidence that an intervention improves a short-term health indicator is also insufficient to establish a system-level benefit if response durability, manufacturing cost and upstream environmental burdens are not considered. Within the proposed framework, functional interventions should therefore be selected according to a diagnosed biological or technological constraint and supported by reproducible evidence under the intended production conditions.

## 3. Efficiency Improvement: Precision Nutrition and Feed Utilization Optimization

### 3.1. Conceptual Framework and Measurement System of Feed Efficiency

Growing global food security pressures, rising feed and production costs, and tightening agricultural carbon reduction regulations have collectively reshaped the connotation of poultry feed efficiency, moving this critical indicator beyond the narrow scope of simple productive performance evaluation. As a multidimensional core metric for modern intensive poultry farming, feed efficiency inherently integrates economic profitability, resource utilization sustainability, and ecological environmental footprints, serving as a fundamental benchmark for high-quality industrial development [135,136].

A suite of complementary indicators enables comprehensive and dimensional quantification of poultry feed efficiency, with each index capturing distinct attributes of nutritional metabolism and resource utilization. The feed conversion ratio (FCR) remains the most classic and universally adopted industrial benchmark, offering intuitive quantification of feed input per unit of finished poultry products and facilitating rapid on-farm productive assessment [137,138,139,140,141,142,143]. The comparative analysis of different interventions on feed conversion ratio is shown in Table 2. Residual feed intake (RFI), by contrast, excludes phenotypic interference from individual growth rate and body size differences, enabling precise evaluation of basal maintenance energy consumption and redundant feed waste [144]. This unique advantage renders RFI a powerful tool for genetic screening and precision breeding of high-efficiency poultry populations [145]. Nutrient utilization efficiency, represented by nitrogen and energy deposition rates, characterizes the transformation capacity of dietary nutrients into edible animal tissues [146]. It bridges in vivo metabolic processes and external environmental outcomes, directly linking nutrient absorption and deposition efficiency with fecal nutrient loss and greenhouse gas emissions, thereby connecting basic nutritional mechanism research with industrial ecological evaluation.

Poultry feed efficiency represents a sophisticated quantitative trait, synergistically modulated by the hierarchical coupling of genetic background, nutritional regimes, rearing management and ambient environmental conditions [147]. Genetic characteristics define the inherent rules of in vivo nutrient partitioning and metabolic operation, setting the theoretical upper limit of feed utilization efficiency and laying a stable germplasm foundation for long-term population performance improvement [148]. Dietary formulation influences nutrient supply and apparent utilization by determining ingredient selection, digestible nutrient balance, anti-nutritional factor load and additive compatibility; intrinsic ingredient properties, processing conditions and intestinal function remain the primary determinants of digestibility [149]. Intestinal physiological status acts as an indispensable prerequisite for efficient nutritional metabolism; intact mucosal barrier structure and stable microecological homeostasis minimize invalid nutrient loss and mitigate metabolic inefficiency induced by chronic subclinical inflammation [150]. Adverse environmental stress further elevates basal maintenance energy demands, pre-empts nutrient allocation for growth and production, and ultimately constrains flock productive potential. Such intricate multi-factor interactions suggest that isolated single-factor optimization can hardly maximize industrial benefits, while systematic and refined nutritional modulation is essential to break the efficiency bottlenecks restricting intensive poultry production.

Comparisons based on FCR require cautious interpretation because an apparent improvement may reflect changes in feed intake, growth rate, mortality-adjustment procedures or physical feed quality rather than greater nutrient utilization alone. Feed form and pellet characteristics are particularly important potential confounders. Pellet durability, fines and hardness can influence feed intake, feeding behaviour and energy expenditure, yet these variables are not consistently reported in nutritional intervention studies. Studies comparing dietary treatments should therefore describe feed form, report relevant pellet-quality measurements and state clearly whether and how FCR was adjusted for mortality.

### 3.2. Precision Amino Acid Nutrition and Ideal Protein Model

Precision amino acid nutrition anchored in the ideal protein model constitutes the most theoretically rigorous, empirically verified and industrially feasible technical pathway for enhancing poultry feed efficiency. This theoretical framework dynamically matches digestible amino acid ratios to the stage-specific physiological metabolism and productive characteristics of poultry, minimizing catabolic waste derived from amino acid imbalance and maximizing whole-body protein deposition efficiency [151,152]. Poultry protein nutrition research has undergone three iterative paradigm transformations, evolving from crude protein-dependent empirical formulation to digestible amino acid-corrected technical systems and ultimately to an integrated framework combining net energy evaluation and ideal protein theory [153]. These progressive innovations have resolved prominent industrial limitations, including unstable ingredient digestibility, excessive amino acid catabolism and mismatched energy–protein ratios, driving poultry protein nutrition from passive empirical adaptation toward active quantitative precision regulation.

Amino acid-balanced low-protein diets can reduce nitrogen excretion and dependence on protein-rich ingredients, but their economic efficiency is context-dependent and often limited by crystalline amino acid prices, reformulation costs and performance risk when crude protein is reduced beyond biologically safe limits [154]. Jiang et al. [155] challenged traditional perceptions of amino acid nutrition and confirmed non-essential amino acids as core functional limiting factors under low-protein feeding regimes. Glycine and serine are crucial for systemic nitrogen transport, intestinal mucosal repair, and purine synthesis in poultry; their endogenous synthesis is insufficient to sustain normal metabolism under low-protein stress, while targeted exogenous supplementation of these amino acids can boost poultry nitrogen utilization efficiency to over 80% [156,157]. Multiple broiler studies across different growth stages have confirmed the beneficial effects of glycine- and serine-based amino acid supplementation on alleviating low-protein diet-induced growth limitations. Ospina-Rojas et al. [156] reported that for 1–21-day-old broilers, the compromised growth performance and serum parameters caused by a 3% crude protein (CP) reduction (22% to 19% CP) could be fully restored by adjusting the digestible Gly+Ser:Lys ratio to 137 or 147. In a follow-up study, Ospina-Rojas et al. [157] demonstrated that glycine supplementation linearly improved weight gain, feed efficiency, intestinal mucin secretion and apparent fat digestibility in 21–35-day-old broilers under low-protein conditions, with combined glycine, serine and threonine supplementation producing superior growth and nutrient digestion benefits. In line with these results, Chrystal et al. [158] verified that co-supplementation of Gly+Ser and threonine enhanced ileal amino acid digestibility in 7–35-day-old broilers fed 165 g/kg CP low-protein diets, normalizing their growth performance to the level of high-protein-fed broilers. For late-stage broilers (29–42 days of age) fed 18% CP low-protein diets, Liu et al. [159] identified 137% as the optimal standardized ileal digestible Gly+Ser:Lys ratio, which completely eliminated low-protein diet adverse effects and achieved equivalent growth performance and nitrogen retention efficiency to normal-protein controls. Further supporting these findings, Dean et al. [160] confirmed that glycine supplementation optimized feed conversion in 1–21-day-old broilers under extreme low-protein stress (16% CP), and a total dietary Gly+Ser level of 2.32% was sufficient to maintain normal growth performance identical to that of standard protein diet groups. Metabolically, excessive amino acid deamination for energy production triggers urea accumulation and oxidative stress, representing a thermodynamically inefficient metabolic pathway [161]. Optimizing dietary energy–protein balance and reducing unnecessary amino acid catabolism therefore underpins the core mechanism of high-efficiency low-protein feeding systems.

Low-protein diets exhibit a non-linear risk profile. Moderate crude-protein reduction, when accompanied by adequate digestible amino acid supply, can improve nitrogen retention and reduce nitrogen excretion without compromising productive performance. Further reductions, however, may expose limitations in glycine equivalents, essential and non-essential amino acids, electrolyte balance, dietary energy and intestinal function. Excessive protein restriction may also impair intestinal mucosal integrity and alter muscular lipid deposition and flavour-precursor metabolism, even when changes in final body weight are less apparent [162]. These constraints differ among genotypes and production stages, making a single degree of protein reduction unsuitable across commercial poultry systems [163]. The economically preferred formulation may differ from the biological optimum because the savings achieved through reduced soybean-meal inclusion depend on crystalline amino acid prices, reformulation costs and the risk of impaired performance or product quality. The objective should therefore be to identify the lowest biologically and economically feasible crude-protein range rather than to minimize crude protein as an independent formulation target.

### 3.3. Feed Enzymes: Unlocking the Untapped Potential of Feed Nutrition

Exogenous enzymes can improve the release and utilization of nutrients that are poorly accessible in conventional feed ingredients. Poultry lack sufficient endogenous enzymatic capacity to hydrolyse several non-starch polysaccharides, phytate complexes and other refractory substrates, thereby limiting the availability of encapsulated nutrients [164]. Appropriately selected exogenous enzymes can partly alleviate these substrate-specific digestive constraints. Phytases, non-starch-polysaccharide-degrading enzymes and proteases target different dietary substrates, and their efficacy depends on matching enzyme specificity and activity to ingredient composition, substrate concentration, feed-processing conditions and the target production stage [165]. Although combinations of enzymes may provide complementary effects, such responses should be demonstrated experimentally rather than assumed from their inclusion in the same preparation.

Phytase stands as the most commercially mature and empirically validated feed enzyme in intensive poultry production. Phytate ubiquitously present in cereals and oilseeds chelates phosphorus, calcium, zinc and other mineral elements to form indigestible macromolecular complexes, triggering massive mineral nutrient waste, excessive inorganic phosphorus supplementation and aggravated fecal phosphorus pollution in traditional feeding systems [166]. Exogenous phytase efficiently hydrolyses stable phytate structures, releases bioavailable bound minerals, reduces redundant inorganic phosphorus input and alleviates water and soil eutrophication risks, comprehensively improving dietary mineral utilization efficiency [167]. Dominated by xylanase and β-glucanase, non-starch polysaccharide enzymes show superior adaptability to wheat and barley-based diets [168]. These enzymes reduce chyme viscosity, dismantle plant cell wall-mediated nutrient encapsulation, and release trapped starch and protein substrates, substantially boosting dietary energy and nutrient digestibility. Protease compensates for insufficient endogenous protease activity in poultry, degrades refractory dietary proteins and improves amino acid bioavailability, effectively expanding the adjustable range of dietary crude protein and underpinning the stable application of low-protein diets.

Enzyme super-dosing strategies, which adopt supplementation levels far exceeding conventional nutritional recommendations, have been progressively popularized in intensive poultry production, integrating feed efficiency improvement and intestinal health modulation into a unified technical system. Enzyme super-dosing may increase the hydrolysis of residual enzyme-sensitive substrates, although the response depends on substrate availability, enzyme recovery after processing, and the dietary matrix [169]. Nevertheless, this strategy carries notable practical trade-offs and application limitations. Super-dosing markedly increases feed formulation costs, while standardized optimal dosage thresholds for different dietary matrices remain undefined. Excess enzyme activity also poses potential risks of degrading intestinal protective mucus layers, triggering mucosal structural damage and disrupting intestinal physiological stability.

### 3.4. Novel Feed Ingredients: Alternative Protein Sources

Soybean meal remains a major poultry-feed protein because of its nutritional value and established supply chain. Its environmental footprint is not uniform but depends on geographical origin, crop yield, farming practices, transportation and, particularly, whether recent land-use change is attributed to its production. Region-specific life cycle assessment is therefore required when comparing soybean meal with alternative protein sources [170,171]. Driven by dual demands for feed efficiency upgrading and industrial low-carbon transformation, the exploration of efficient, low-carbon and sustainable alternative protein sources has evolved into a core interdisciplinary research direction. Various alternative protein materials, namely insect protein, microalgal protein, single-cell protein, oilseed by-products and agro-industrial residues, each feature distinctive resource advantages and great industrialization prospects [172,173,174,175,176,177,178,179]. A comprehensive comparison of these diverse alternative protein sources under commercial production conditions is presented in Table 3.

Different novel protein sources deliver differentiated nutritional supplementation and ecological benefits for modern poultry production. Black soldier fly larvae protein features a well-balanced amino acid profile rich in limiting amino acids, capable of optimizing poultry growth performance and feed conversion efficiency while realizing high-value recycling of organic waste biomass [172]. Microalgae such as spirulina and azolla are abundant in essential amino acids, unsaturated fatty acids and diverse bioactive compounds, achieving integrated effects of nutritional fortification and immune modulation [180]. Lobo et al. [181] verified via dual-flow in vitro rumen culture that 50% partial replacement of soybean meal with microalgae biomass lowered ruminal protein degradation markers and improved nitrogen utilization efficiency, with Chlorella pyrenoidosa showing stronger rumen bypass protein performance than Spirulina platensis. Single-cell protein manufactured via precision fermentation is decoupled from arable land, seasonal cycles and climatic risks that restrict conventional crop protein production, delivering ultra-high productivity and consistent nutritional profiles [182]. Double-low rapeseed meal can partially replace soybean meal where regional supply and diet formulation permit, although differences in digestible amino acid content, fibre and residual anti-nutritional factors must be considered [183]. Its GHG advantage is not inherent but depends on crop yield, processing energy, transport distance, dietary supplementation and the allocation of environmental burdens between rapeseed oil and meal. Grape and olive pomace may similarly support regional waste valorization and supply fibre and polyphenols, but their nutritional and productive effects vary with source, processing and inclusion level [184,185]. Diet-level, region-specific LCA is therefore required to establish whether these ingredients provide a net environmental benefit.

Before large-scale inclusion in poultry diets, novel protein ingredients should progress through a stage-gated assessment covering commercial supply, safety, nutritional equivalence and whole-system performance. Commercial supply is the primary constraint on adoption by modern feed mills and therefore constitutes the initial screening gate. Annual production capacity, continuity of delivery, geographical concentration, batch-to-batch consistency and traceability must be compatible with industrial throughput, storage capacity and quality-control requirements. Ingredients available only in small or seasonally variable quantities may remain valuable in local or small-scale feeding systems, but they cannot be assumed to be scalable substitutes for conventional protein sources. The subsequent gate concerns biological and chemical safety. Rearing or cultivation substrates, microbial contamination, mycotoxins, heavy metals, pesticide or veterinary-drug residues and source-specific anti-nutritional factors require validated analytical limits. In insect meals, chitin concentration, microbial load and contaminants transferred from rearing substrates are important variables; in microalgal ingredients, resistant cell walls, mineral accumulation and species-specific toxins require particular attention. The term “detoxification” should be used only when the target hazard, removal or inactivation procedure, analytical detection method and acceptable residual-risk threshold are clearly defined. For example, biological processing can reduce specific anti-nutritional factors in rapeseed meal, although its efficacy and the resulting residual risk depend on the processing method [186].

The third gate is processing and nutritional equivalence at the complete-diet level. Drying, defatting, cell disruption, extraction and decontamination may improve stability or nutrient accessibility but can also increase energy demand, production cost and heat-related nutrient damage. Feeding trials should therefore determine digestible amino acid and metabolizable energy values, palatability, feed intake, pellet quality and the additional amino acid or enzyme supplementation required to maintain productive performance. The fourth gate comprises long-term physiological and life cycle validation. This should include, where relevant, multi-cycle assessments of health and metabolism, manufacturing yield, processing energy, transportation, coproduct allocation, supply-chain losses and sensitivity to production scale. Market acceptance represents an additional deployment constraint, particularly where consumers associate insect-derived or waste-based feed ingredients with product safety or quality; however, these perceptions are likely to vary across regions and market segments. A feed ingredient should not be classified as low-carbon solely because it originates from waste valorization, insects, algae or microbial fermentation. Such a claim requires a stable commercial supply chain and a complete-diet LCA demonstrating a net environmental benefit under realistic processing, transport and inclusion conditions.

### 3.5. Precision Feeding Technology and Digital Nutrition

Digital technologies can support precision nutritional management by combining flock-, house- or pen-level measurements with environmental data [187,188,189]. Commercial sensors commonly record aggregate feed and water consumption, body-weight distributions, temperature, humidity and air quality, allowing feeding programmes to be adjusted according to production stage and observed flock responses. Individual-bird monitoring generally requires specialized identification and tracking systems and is not yet routine commercial practice. Integrated sensing and intelligent control systems have proven effective for diverse poultry production [190,191,192]. Automated precision feeding stations with built-in weighing units align real-time breeder body weights to target growth curves, lowering flock weight variation coefficient below 2% and boosting uniformity [190]. For broilers, microphone-based acoustic monitoring paired with a pecking detection algorithm achieves 86% feed intake prediction accuracy, with an R^2^ of 0.994 relative to physical weighing records [191]. An online weighing-feeding system controlled via an adaptive compact dynamic process model regulates broiler growth trajectories with only 3.7–6.0% average relative error, likewise improving flock homogeneity [192]. Together, these studies confirm that real-time phenotypic monitoring coupled with dedicated algorithms optimizes feeding strategies and standardizes growth performance across poultry varieties. Nutrigenomic approaches can identify strain-specific nutrient requirements and metabolic responses, thereby supporting genotype-informed formulation [193]. Big data and artificial intelligence integrate long-term multi-dimensional breeding datasets, realizing accurate growth curve prediction, stage-specific formula intelligent optimization, and early warning of stress and disease risks through abnormal data fluctuations, which minimizes invalid nutritional consumption and resource waste [194].

Digital precision-feeding models should be considered commercially deployable only after external validation across independent farms, poultry breeds, production stages, seasons, feed forms and sensor platforms. Development datasets must represent the intended application domain, and all observations from the same farm or production cycle should be assigned exclusively to either model development or external testing to prevent information leakage. Model evaluation should report calibration as well as predictive discrimination, together with performance under missing-data, sensor-failure and out-of-distribution conditions. Operational systems should incorporate drift detection, uncertainty estimates and a predefined safe fallback feeding rule when predictions fall outside the validated domain. Transfer learning and hierarchical modelling may reduce the amount of local calibration data required, but they do not replace prospective validation under commercial conditions. Studies should report the loss of predictive accuracy after transfer, the volume of local data required for recalibration, and whether the economic benefit remains after accounting for hardware, maintenance, connectivity and da-ta-management costs. Until these requirements are met, claims of cross-farm scalability should remain conditional.

## 4. Low-Carbon Pathways: Nutritional Strategies for Greenhouse Gas Emission Mitigation

### 4.1. Source Analysis of Carbon Footprints in Poultry Production

Across global livestock industries, redefining the theoretical and practical boundaries of agricultural GHG mitigation [195]. Conventional mitigation practices reliant exclusively on terminal manure remediation are being progressively superseded by full-value-chain regulatory paradigms that integrate feed cultivation, in vivo animal metabolism and post-production waste cycling [196]. As the most resource-efficient and scalable terrestrial protein production system, intensive poultry farming demands rigorous mechanistic clarification of its GHG emission signatures, sectoral contribution partitioning and core metabolic drivers [197]. Elucidation of these foundational mechanisms underpins the development of targeted nutritional mitigation frameworks and facilitates the sustainable low-carbon transition of commercial poultry production. Using LCA, poultry GHG emissions fall into three linked sources including feed production, enteric fermentation and manure management, each with unique substrates, gas makeup and carbon budget impacts [198].

Feed production constitutes the dominant carbon source of poultry industrial systems, accounting for over 60% of total anthropogenic GHG emissions and harbouring the greatest untapped mitigation potential [199]. Agrochemical fertilization during feed crop cultivation activates soil nitrification and denitrification cascades, releasing nitrous oxide (N_2_O), a high-potency greenhouse gas with profound radiative forcing effects [200]. Cropland expansion for staple feed commodities including maize and soybean disrupts native soil carbon reservoirs, triggering organic carbon mineralization and net carbon dioxide (CO_2_) efflux [201]. Fossil fuel combustion throughout feed processing, long-distance transportation and mechanical pelleting further exacerbates supply-chain carbon accumulation [202]. Unlike ruminant livestock, poultry exhibit simplified digestive architectures and negligible intestinal methanogenic abundance, rendering individual enteric methane (CH_4_) emissions physiologically trivial [203]. The extraordinary scale of global poultry population nevertheless aggregates these minimal individual fluxes into climate-altering emission magnitudes [204]. Manure storage, stacking and field incorporation represent critical terminal emission hotspots. Sustained anaerobic microbial metabolism drives continuous CH_4_ generation, while fluctuating oxic–anoxic redox conditions recurrently stimulate N_2_O efflux and ammonia (NH_3_) volatilization [205]. Atmospheric redeposition of reactive nitrogen compounds further propagates indirect GHG forcing and cascading ecosystem degradation [206].

Poultry production possesses inherent low-carbon attributes relative to swine and ruminant farming systems, characterized by markedly reduced emission intensity per unit edible product. Ongoing global intensification and industrial expansion of poultry operations, however, have substantially elevated overall breeding throughput, resulting in net growth in sectoral GHG output [203,204]. Pure intensity-based emission reduction can no longer accommodate the stringent carbon constraints governing modern sustainable agriculture. Mechanistic breakthroughs beyond conventional end-of-pipe engineering mitigation are therefore imperative. Precision nutritional modulation, which optimizes systemic nutrient retention, eliminates redundant metabolic nitrogen accumulation, and remodels whole-organism emission phenotypes, provides a robust, actionable pathway for long-term, stable decarbonization of the poultry industry.

### 4.2. Emission Reduction Effects of Low-Protein Diets

Amino acid-balanced low-protein diets, formulated according to the ideal protein concept, represent a practical strategy for improving nitrogen-use efficiency in poultry production [207,208]. Supplementation with crystalline amino acids allows moderate reductions in dietary crude protein while maintaining the supply of limiting amino acids, although the feasible degree of protein reduction depends on genotype, production stage, dietary energy, glycine equivalents and the balance of essential and non-essential amino acids. Hernández et al. [207] evaluated diets in which crude protein was reduced by 1.5 or 3.0 percentage points across a four-phase feeding programme for male and female broilers from 1 to 48 days of age. Nitrogen excretion was reduced by 9.5% and 17.0% in males and by 11.8% and 14.6% in females, respectively. Nanto-Hara et al. [208] similarly evaluated amino acid-balanced low-protein diets in male broilers from 1 to 50 days of age and estimated an approximately 31.1% reduction in cumulative nitrogen excretion relative to the higher-protein control. Reducing nitrogen excretion can limit the substrate available for NH_3_ volatilization and direct or indirect N_2_O formation during manure storage, treatment and land application [208,209]. The magnitude of these effects nevertheless depends on manure characteristics and management conditions and should not be inferred solely from dietary nitrogen intake [210,211].

Lower nitrogen excretion and improved nitrogen-use efficiency do not necessarily produce an equivalent reduction in the whole-chain carbon footprint. The manufacture of crystalline amino acids, particularly fermentation-derived lysine and threonine, requires feedstocks and energy for aeration, separation, concentration and drying, thereby generating upstream environmental burdens [37,38,212]. Conversely, reducing soybean-meal inclusion may lower diet-related impacts in supply chains where soybean production is associated with substantial agricultural inputs, long-distance transport or recent land-use change. These effects are not uniform because the environmental footprint of soybean meal varies with geographical origin, crop yield, farming practices, land-use-change assumptions and coproduct allocation [213,214,215,216,217,218,219]. The net outcome therefore depends on the amount and source of soybean meal displaced, the types and quantities of supplemental amino acids, manufacturing efficiency, regional energy mix, transport and animal performance. Claims of GHG mitigation should consequently be supported by diet-level LCA that includes amino acid manufacture and relevant changes in ingredient sourcing, rather than inferred from reduced nitrogen excretion alone. The influence of alternative land-use-change assumptions on the estimated footprint of soybean meal is summarized in Table 4.

Industrial scaling of low-protein diet technology is nonetheless constrained by definable physiological thresholds, unresolved methodological gaps and context-dependent ecological–economic trade-offs. A unified LCA specification tailored exclusively to poultry production systems remains absent, with existing studies adopting heterogeneous functional units including feed mass, live weight gain, finished product yield and complete breeding cycles. Inconsistent evaluation frameworks undermine cross-study comparability and hinder the establishment of unified sectoral technical standards. Dietary crude-protein reduction is further bounded by intrinsic physiological limitations. Excessive protein restriction impairs intestinal mucosal anabolism, destabilizes microecological homeostasis, suppresses growth performance and remodels muscle lipid and flavour metabolism, ultimately deteriorating poultry product quality [220]. Compensatory feed intake elevation to offset productivity loss increases upstream supply-chain carbon emissions, fully neutralizing on-farm environmental gains and creating dynamic antagonisms between economic profitability and ecological sustainability [221].

### 4.3. Environmental Benefits of Functional Feed Additives

Functional feed additives, encompassing probiotics, exogenous enzymes and plant-derived bioactive metabolites, are conventionally deployed to sustain intestinal physiological integrity, improve feed conversion efficiency and substitute antibiotic growth promoters in intensive poultry farming [50,51]. Their latent capacity to modulate agricultural GHG emissions has emerged as a high-priority interdisciplinary frontier bridging animal nutritional science and climate sustainability research. Distinct from dedicated engineering-based mitigation technologies, additive-associated decarbonization arises from secondary synergistic metabolic regulation rather than direct emission suppression [222]. These bioactive compounds reshape whole-industry carbon budgets by refining nutrient metabolic efficiency, minimizing unproductive nutrient dissipation and elevating overall resource utilization, thereby unifying productivity enhancement and low-carbon governance via two complementary mechanistic axes [223].

Functional additives mitigate upstream supply-chain carbon emissions by enhancing feed digestibility and reducing unit-product feed consumption [224]. Compound non-starch polysaccharide enzymes, dominated by xylanase and β-glucanase, degrade recalcitrant plant cell wall matrices, reduce gastrointestinal digesta viscosity, and liberate encapsulated nutrients, increasing dietary energy utilization efficiency by approximately 8% [225]. Probiotics and plant bioactive compounds alleviate production-associated stress responses, stabilize intestinal microecological equilibrium and reduce inflammatory metabolic waste accumulation, further optimizing systemic feed efficiency [50,51,226]. Equivalent productive output with diminished feed input directly curbs CO_2_ emissions originating from feed cultivation, industrial processing and cross-regional transportation [226]. These additives concurrently attenuate terminal manure GHG emissions by improving dietary nitrogen and phosphorus bioavailability. Exogenous protease augments dietary protein degradation and intestinal absorption, phytase mobilizes bound phosphorus reserves, and probiotics rebalance hindgut nitrogen metabolic flux [227]. Synergistic functional modulation substantially reduces fecal nitrogen and phosphorus excretion, limiting substrate availability for manure N_2_O and NH_3_ generation and enabling simultaneous pollutant remediation and GHG abatement [227,228].

Current understanding of additive-derived low-carbon benefits is constrained by systematic evaluation biases and practical application trade-offs. Most existing investigations prioritize short-term on-farm emission metrics while neglecting embodied carbon costs incurred during additive microbial fermentation, purification, industrial manufacturing and logistics. A holistic “production–application–disposal” full life cycle evaluation framework remains unestablished for functional feed additives. Certain high-value compound additives carry substantial manufacturing carbon footprints, such that on-farm mitigation gains can be fully offset by upstream carbon expenditure, eliminating net climatic benefits. Standardized LCA protocols, rather than localized single-point monitoring, are therefore essential to objectively quantify the authentic low-carbon efficacy of functional additive-based nutritional strategies.

### 4.4. Low-Carbon Feed Ingredients

Feed ingredients are a major determinant of the carbon footprint of poultry production. This section evaluates three potential mitigation pathways: alternative protein sources, regionally available feed ingredients and agro-industrial coproducts. Their environmental benefits are treated as context-dependent and require assessment of processing, transport, nutritional equivalence and diet-level performance.

Alternative protein sources may reduce reliance on soybean meal, but their environmental performance varies with production route, processing requirements, transport, nutrient density and the supplementation needed to maintain complete-diet performance [172,229,230,231]. Regionally available ingredients and agro-industrial coproducts may reduce transport or waste-management burdens in specific supply chains, although local origin or coproduct status does not establish an inherent GHG advantage [232,233]. Comparisons should therefore be based on region-specific, diet-level LCA that accounts for ingredient processing, coproduct allocation, transport and productive response.

Scaled industrial application of low-carbon feed ingredients is hindered by inherent operational bottlenecks and economic–environmental trade-offs. Novel protein biomasses such as insect larvae and microalgae face high manufacturing costs and immature supply chain infrastructures, elevating economic input for commercial poultry operations. Common agricultural by-products, including rapeseed meal, contain endogenous anti-nutritional metabolites exemplified by glucosinolates; inappropriate inclusion levels impair gastrointestinal nutrient digestion and absorption, offsetting the intrinsic low-carbon advantages of these raw materials [234]. Severe seasonal output volatility and insufficient supply stability further restrict the large-scale deployment of niche by-products in standardized intensive production systems, imposing definitive scenario-specific application boundaries.

### 4.5. Nutritional Correlation of Manure GHG Mitigation

Traditional research paradigms artificially compartmentalize manure GHG mitigation as a standalone environmental engineering challenge, decoupling terminal waste emission dynamics from upstream dietary nutritional regulation mechanisms [235]. In practice, feed nutritional profiles act as a primary deterministic factor governing fecal nitrogen and phosphorus concentrations, organic matter structural characteristics, and post-excretion microbial community assembly, collectively defining the inherent GHG emission potential of manure [171,236]. Source-oriented nutritional intervention functionally complements and amplifies terminal engineering remediation technologies, providing critical mechanistic support for in-depth on-farm emission abatement.

Precision dietary modulation suppresses GHG substrate biogenesis at the production source. Low-protein and low-available-phosphorus feeding regimens reduce fecal nutrient excretion, limiting substrate availability for anaerobic fermentation and nitrification–denitrification reactions and fundamentally attenuating manure CH_4_ and N_2_O emission capacity [237]. Targeted nutritional reshaping remodels fecal microbial ecology; dietary probiotic supplementation stabilizes intestinal flora homeostasis, with persistent microbial legacy effects inhibiting the proliferation of methanogens and nitrifying bacteria in excreted manure and slowing GHG release kinetics [238]. Nutritional strategies can be synergistically integrated with litter modification to amplify mitigation efficacy [239]. Combined diet optimization and biochar-amended bedding leverage the porous microstructure of biochar to adsorb free NH_3_ and suppress nitrifying microbial activity, reducing field-scale manure-derived N_2_O emissions by 22% [239,240]. This integrated nutrition–engineering system achieves dual-layer, synergistic GHG abatement.

Nutritional source control nevertheless exhibits distinct mitigation ceilings. It modulates manure emission potential by reshaping nutrient composition and metabolic properties but cannot replace terminal engineering interventions including sealed storage, aerobic composting and biogas residue recycling. Nutritional source regulation and end-of-pipe engineering management are functionally complementary rather than substitutive, with their synergistic integration representing the optimal approach to maximize whole-process manure mitigation efficiency.

### 4.6. Nutritional Matching for Low-Emission Strain Breeding

Genetic improvement and precision nutrition represent complementary approaches to increasing the resource efficiency of poultry production. Genetic background influences growth potential, nutrient partitioning and feed efficiency, whereas dietary conditions determine the extent to which these traits are expressed. Genetic improvement in feed efficiency can reduce the feed required and the associated nutrient losses per unit of marketable poultry product, thereby potentially lowering the environmental footprint of production [4,241]. These benefits should nevertheless be evaluated at the production-system level because selection for rapid growth or feed efficiency does not necessarily improve health, product quality or environmental performance under all dietary and climatic conditions [242,243].

Genotype-specific nutritional responses may influence the extent to which improvements in feed efficiency translate into lower environmental burdens. Available studies demonstrate that broiler genotypes can differ in their responses to dietary protein, lysine and methionine supply [244,245], but these findings should not be interpreted as evidence for a distinct class of “low-emission genotypes”. Traditional extensive unified feeding systems fail to accommodate strain-specific metabolic signatures, masking the inherent low-carbon advantages of elite genotypes and limiting breeding-derived mitigation gains. Stage-specific, genotype-customized nutritional regimens enable precise gene–nutrition matching and maximize the climatic benefits of genetic improvement. Gene–nutrition interactions should be interpreted as genotype × diet × environment responses rather than fixed effects of individual nutrients. Dietary amino acids and energy availability are sensed through mTORC1, GCN2 and AMPK, whereas oxidative and xenobiotic signals converge on Nrf2–Keap1, AhR and related stress-response networks. These pathways regulate translation, mitochondrial substrate use, antioxidant defence, epithelial turnover and immune allocation. Genotype-dependent variation in nutrient transporters, receptor sensitivity, enzyme activity and regulatory sequence architecture can therefore alter the magnitude and direction of a phenotypic response to the same diet. Microbial metabolites add a further layer by engaging host receptors and by changing substrates for histone acetylation, DNA methylation and non-coding-RNA regulation. These mechanisms provide plausible routes through which nutrition modifies phenotypic penetrance, but pathway activation alone should not be treated as proof of a causal gene–nutrient mechanism. Environmental stress can expose otherwise latent genotype-specific responses. Heat stress, pathogen pressure and high stocking density increase corticosterone signalling, oxidative load, intestinal permeability and maintenance energy demand; these changes redistribute nutrients away from growth or egg production and can modify AMPK–mTOR and inflammatory signalling. A genotype that performs efficiently under thermoneutral conditions may therefore lose its advantage when the same diet is applied during heat or immune challenge. Future studies should use factorial genotype × diet × stress designs, longitudinal phenotyping and independent commercial validation. Candidate mechanisms require allele- or haplotype-resolved expression, epigenomic or chromatin-accessibility measurements, protein and metabolic-flux confirmation, and, where feasible, pathway perturbation. This design would distinguish true modification of genotype-specific phenotypic penetrance from non-specific stress adaptation. Collectively, resolving these genotype × diet × environment interactions will be essential for developing robust genotype-specific nutritional models that retain their productive and environmental benefits under commercial stress conditions.

Across the strategies considered in this section, evidence for nutritional GHG mitigation remains sensitive to system boundaries, baseline comparators and modelling assumptions. Reduced manure nitrogen, improved feed efficiency or the use of alternative ingredients does not alone establish a lower carbon footprint if upstream amino acid and additive manufacture, ingredient processing, transport or losses in productive performance are excluded. Nutritional strategies should therefore be compared using diet-level biological responses and whole-chain environmental impacts, accompanied by sensitivity analyses for ingredient sourcing, energy supply, land-use change and coproduct allocation [243].

## 5. Synergies, Trade-Offs and Integrated Evaluation

### 5.1. The Central Coupling Mechanism

**Figure 1:**
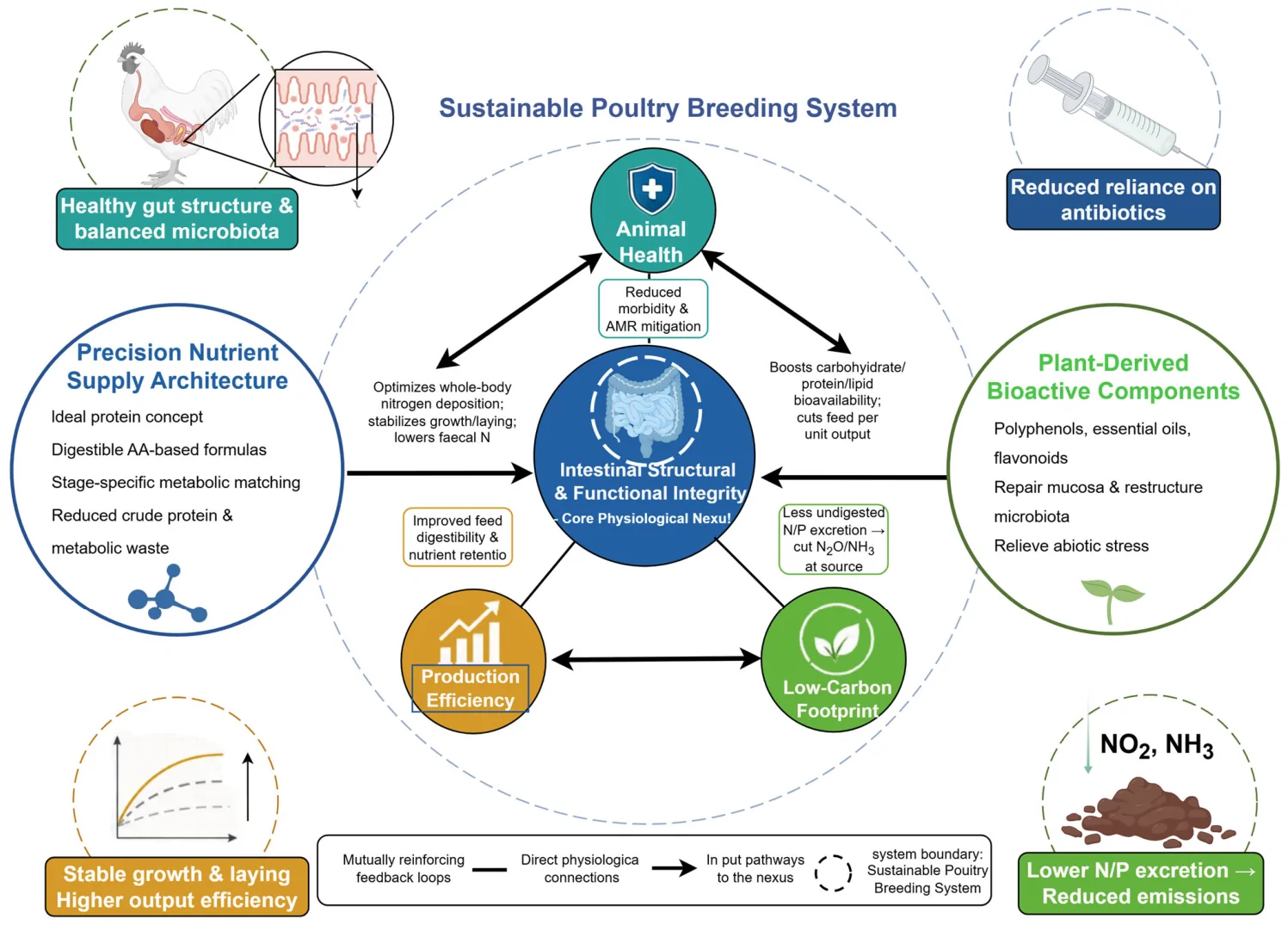
Schematic representation of the synergies and trade-offs among animal health, production efficiency and low-carbon performance in poultry nutrition.

Health, feed efficiency and environmental performance are not independent outcomes. Their most direct common physiological link is nutrient retention [246,247]. An intact intestinal barrier and a stable gut microbiota support nutrient digestion and absorption while limiting the maintenance costs associated with persistent inflammation [248,249,250,251]. Improved nutrient retention can reduce the feed required per unit of poultry product and decrease the quantities of undigested nitrogen and phosphorus entering manure, thereby limiting the substrates available for ammonia, nitrous oxide and downstream nutrient losses [252,253,254]. Conversely, intestinal dysfunction increases nutrient loss, disease susceptibility and reliance on therapeutic interventions [255,256]. These relationships explain why interventions that improve intestinal function may generate productive and environmental co-benefits. However, a health-promoting intervention should not be classified as low-carbon on the basis of its physiological effects alone. Upstream manufacturing burdens, effective inclusion rate, response consistency and changes in whole-cycle productive performance must also be considered. As illustrated in Figure 1, nutrient retention provides a common physiological link among animal health, production efficiency and environmental performance, although interventions targeting one objective may generate benefits or trade-offs in the others.

### 5.2. Where Trade-Offs Arise

Trade-offs emerge when improvement in one objective shifts costs or environmental burdens elsewhere. Excessive crude-protein reduction can lower nitrogen excretion but impair growth, carcass yield or product quality when essential and non-essential amino acid supply is inadequate [257,258,259]. Alternative proteins may reduce dependence on conventional protein sources yet increase diet cost, processing energy or supplementary nutrient requirements. Precision-feeding systems can improve nutrient matching but require capital investment, reliable sensors and data infrastructure. Functional additives may strengthen flock resilience, although variation in product composition and biological response reduces the certainty of their productive and environmental returns. These examples indicate that a nutritional strategy should be evaluated according to its whole-diet and whole-system effects rather than a favourable change in a single indicator.

Product quality should therefore be treated as a formal constraint within low-carbon diet formulation rather than evaluated only after the diet with the lowest estimated environmental impact has been selected [260,261,262,263]. Crude-protein reduction should not proceed solely until detectable growth depression occurs, because changes in muscle protein turnover, intramuscular lipid deposition, water-holding capacity and flavour-precursor metabolism may arise before final body weight is affected. Formulation studies should characterize response surfaces across crude protein and digestible amino acid supply while simultaneously monitoring breast yield, post-mortem pH decline, drip and cooking losses, shear force, intramuscular fat, and key flavour-related amino acids and nucleotides. Glycine equivalents, methionine, threonine, lysine, branched-chain amino acids, electrolyte balance and dietary energy should be evaluated jointly because deficiencies or imbalances can alter nutrient partitioning even when overall growth performance is maintained.

Diet selection should consequently follow a multi-objective decision rule. The preferred formulation is the diet with the lowest verified life cycle environmental burden that remains within prespecified non-inferiority margins for growth, mortality, carcass yield and priority product-quality traits, while retaining economic feasibility. The relative weighting of these outcomes should reflect the production system and target market. Commodity broiler production may prioritize feed efficiency and breast yield, whereas premium poultry markets may assign greater value to tenderness, juiciness and characteristic flavour. Laying systems require separate consideration of egg mass, shell quality and internal egg quality. Sensory evaluation and, where market claims are proposed, willingness-to-pay analysis are needed before concluding that an environmental benefit is compatible with consumer acceptance. Product quality is thus incorporated into the framework as a measurable boundary condition rather than treated as a secondary caveat.

### 5.3. A Hierarchical Decision Framework

**Figure 2:**
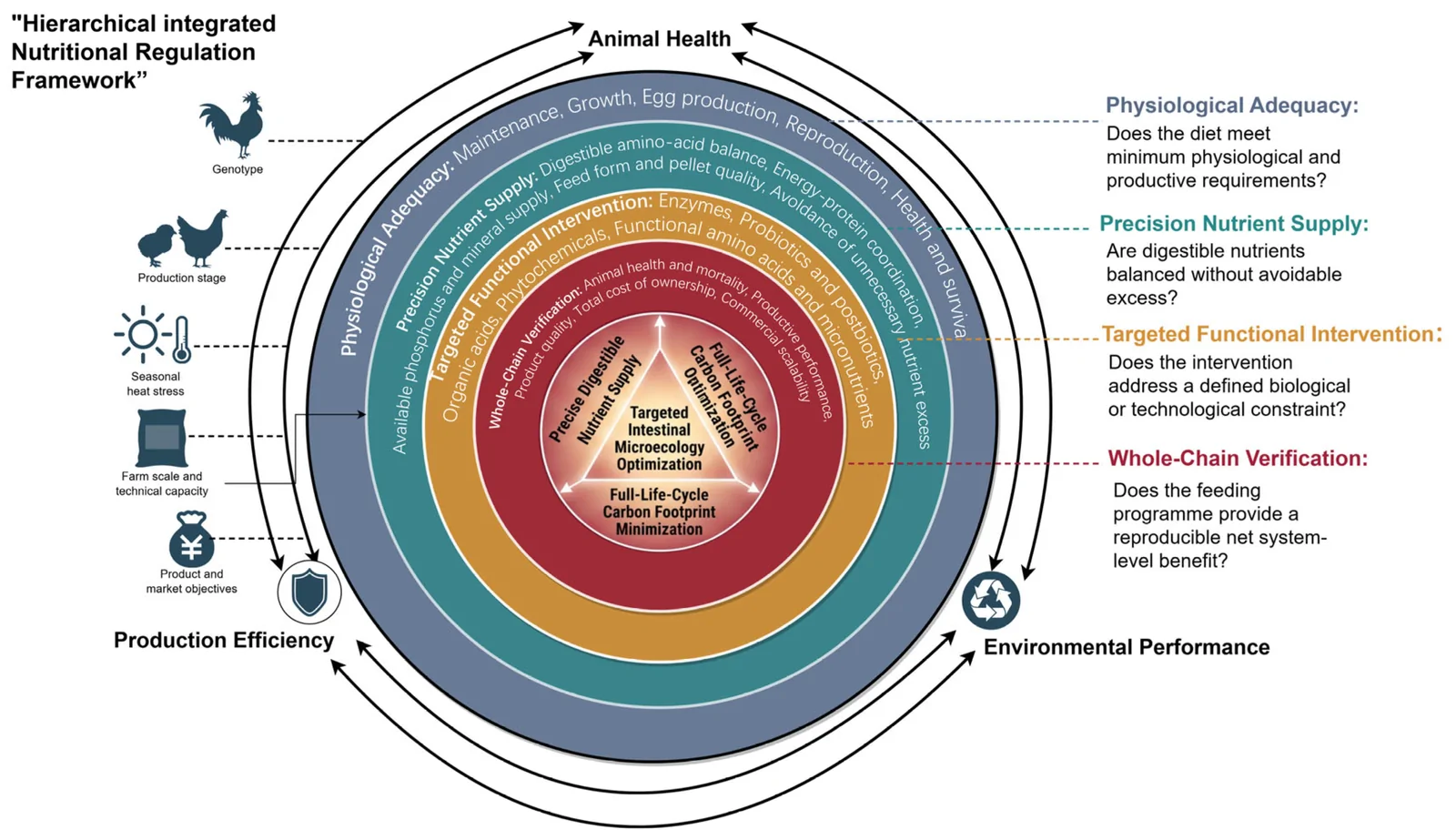
Hierarchical decision framework for the integrated evaluation of poultry nutritional strategies. The framework progresses from physiological adequacy and precision nutrient supply to targeted functional intervention and whole-chain verification. Decisions at each level are jointly constrained by animal health, productive performance, product quality, economic feasibility and environmental impact, while genotype, production stage, environmental stress, ingredient availability and market objectives determine context-specific implementation. Whole-chain assessment provides feedback for iterative dietary optimization.

A practical framework should begin by ensuring that dietary interventions satisfy the nutrient requirements for maintenance, growth, egg production and reproduction, since strategies that compromise these fundamental functions cannot be considered sustainable. Within this physiological boundary, balancing digestible amino acids, energy, and minerals can limit unnecessary nutrient inputs while preserving productive performance and product quality. Enzymes, probiotics, organic acids, phytochemicals and other functional interventions should be incorporated only where a clearly defined biological or technological constraint exists and where the expected response is reproducible under the intended production conditions. The resulting feeding programme must then be assessed across the supply chain, encompassing ingredient production, additive manufacture, feed processing and transportation, animal performance, mortality and manure management. The importance assigned to animal health, production cost, product quality and environmental impact will inevitably differ according to genotype, production stage, disease pressure, regional ingredient availability and market objectives. Rather than prescribing a universal dietary formula, the framework provides a context-dependent basis for evaluating and selecting nutritional strategies (Figure 2).

Commercial adoption should be assessed using total cost of ownership and marginal abatement cost rather than ingredient or equipment price alone. Capital expenditure, maintenance, training, connectivity, formulation changes, response variability and avoided feed or medication costs should be annualized at the farm level. Reported percentage increases in production cost should not be treated as universal because they depend on the technology bundle, farm scale and production context. A tiered implementation pathway may therefore be more realistic, beginning with nutrient balancing and feed-quality control, followed by targeted additives or regional coproducts where a defined constraint and reliable supply chain exist, and progressing to sensor-rich adaptive systems only where scale, margins and technical capacity justify them. Cooperative purchasing, shared advisory services and outcome-based environmental incentives may improve access for small- and medium-sized producers, but their effectiveness requires empirical evaluation.

### 5.4. LCA as the Verification Layer

Life cycle assessment provides the verification layer for the proposed framework by determining whether an apparent on-farm benefit is retained across the wider production system or offset by burdens arising upstream or downstream. The assessment should follow the principles and requirements established in ISO 14040 and ISO 14044, supplemented where appropriate by product-specific Environmental Footprint guidance [264,265,266]. The functional unit should reflect the production objective: broiler assessments may use one kilogram of live-weight gain or carcass weight at the farm gate, whereas laying-hen assessments should preferably use one kilogram of marketable egg mass. Units based on edible protein may support comparisons among production systems but should not replace product-specific functional units within poultry categories. The system boundary should include feed-crop production, land-use change where relevant, ingredient and additive manufacture, feed processing, packaging and transport, animal performance, mortality, and manure storage and management. Because animal health is not adequately represented by a single conventional environmental impact category, morbidity, mortality, medication use, and product losses should be reported as complementary indicators or incorporated through clearly defined scenarios. Comparisons also require sensitivity analyses covering allocation method, geographical sourcing, energy mix, land-use assumptions and variation in productive response. The minimum methodological and reporting requirements proposed for LCA of poultry nutritional strategies are summarized in Table 5.

Standardizing these methodological choices would improve comparability among poultry nutrition LCAs, although harmonization should not obscure genuine variation arising from regional production systems, ingredient sourcing and energy infrastructure. Results should therefore be reported using a common methodological core accompanied by region-specific inventory data and sensitivity analyses. Within the proposed framework, a nutritional strategy should be regarded as providing an integrated net benefit only when it maintains animal health and productive function, remains economically feasible, and reduces environmental burdens without transferring disproportionate impacts to another life cycle stage or environmental category.

## 6. Future Research Priorities

Future research should define the conditions under which nutritional interventions remain effective in commercial poultry production. Responses observed in controlled experiments may vary with genotype, age, basal diet, health status, environmental stress and farm management. Multi-site studies should therefore combine pen- or house-level performance with mortality, medication use, feed quality and relevant environmental measurements. Dose–response relationships should be evaluated across biologically meaningful ranges, allowing recommendations to be expressed as context-dependent operating boundaries rather than universal inclusion levels.

Mechanistic research should explain variation in nutrient retention, stress resilience, productive performance and emission-related traits rather than merely catalogue molecular changes. Longitudinal multi-omics studies, particularly genotype × diet × environment designs, should begin with predefined hypotheses and validate candidate pathways or biomarkers in independent commercial populations. Associations identified through microbiome, transcriptomic or metabolomic analyses require functional confirmation and should ultimately be linked to complete-diet performance and manure nutrient outputs.

Commercial scalability should be evaluated alongside biological efficacy. Emerging proteins, postbiotics and digital feeding systems require evidence of stable supply, processing compatibility, safety, reproducibility and economic feasibility at commercially relevant scales. Feed resources suitable for local production may not meet the volume and consistency requirements of modern feed mills, while digital models validated within a single farm or production cycle cannot be assumed to generalize across breeds, seasons or management systems. External validation should therefore precede claims of industrial applicability.

Environmental and economic assessments should be incorporated during technology development. Prospective LCA must include ingredient and additive manufacture, feed processing, transport, animal performance and manure management, accompanied by sensitivity analyses for sourcing, energy use, allocation and biological response. Comparable reporting of diet composition, feed quality, experimental units, functional units and system boundaries is equally important. Decision-ready evidence should integrate biological efficacy, commercial reproducibility, product-quality protection, economic feasibility and whole-chain environmental performance, enabling poultry nutrition to progress from promising interventions towards verifiable strategies for healthy, efficient and lower-carbon production.

## 7. Conclusions

**Figure 3:**
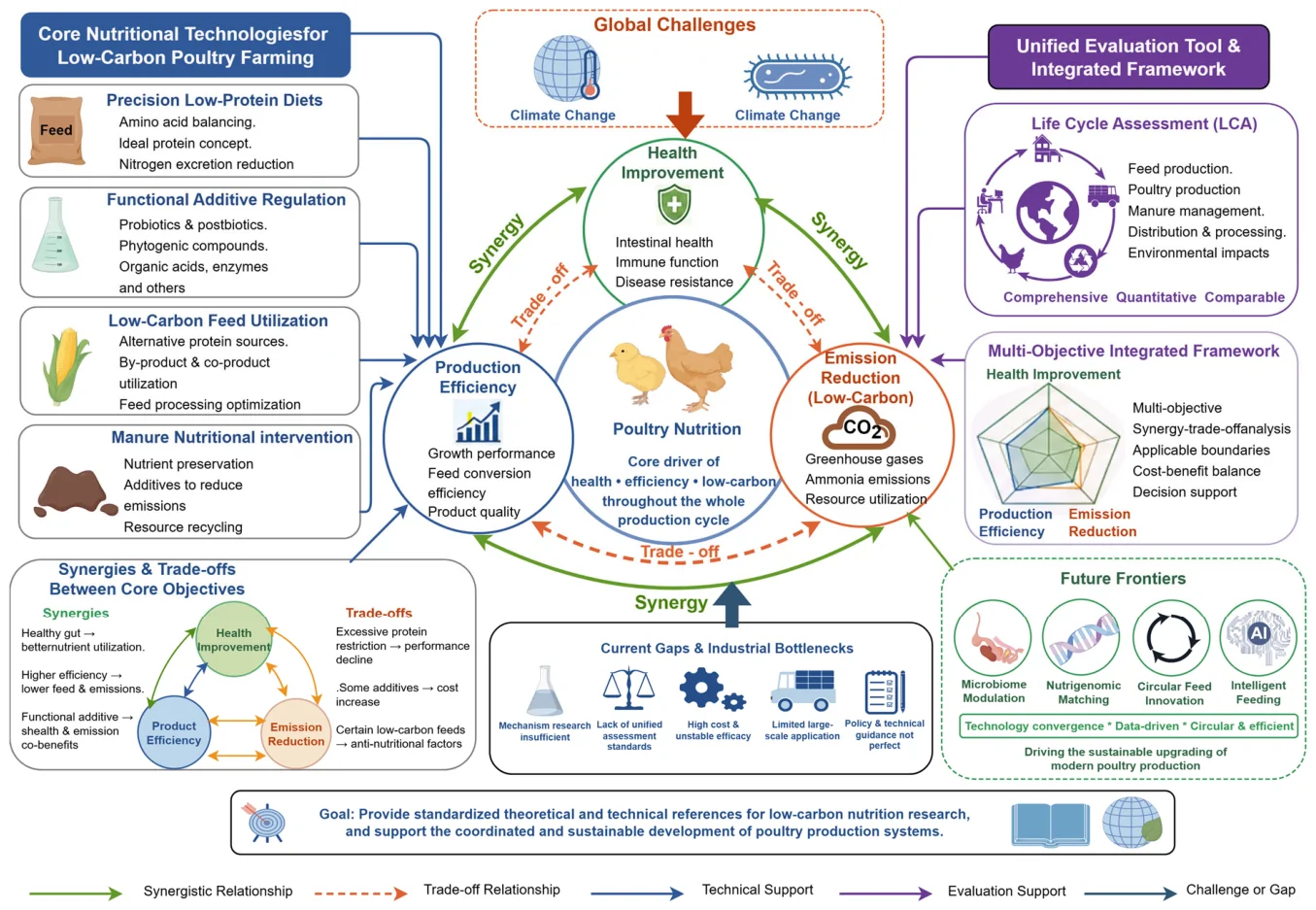
Mechanistic interrelationship among production efficiency, intestinal health and emission reduction guided by low-carbon poultry nutrition.

Poultry nutrition links animal health, productive efficiency and environmental performance through its effects on intestinal function and nutrient retention. More precise nutrient supply can reduce feed requirements and nutrient excretion, but improvements in one outcome may create biological, economic or environmental trade-offs elsewhere. Excessive protein restriction may impair performance or product quality, additives may respond inconsistently under commercial conditions, and alternative ingredients may be limited by processing requirements and supply. Farm-level improvements also cannot establish a lower carbon footprint when upstream ingredient and additive manufacture, transportation or performance losses are excluded. The proposed framework evaluates nutritional strategies through physiological adequacy, precision nutrient supply, targeted functional intervention and whole-chain verification. Health, performance, product quality, economic feasibility and environmental impact are considered jointly, with application conditions determined by genotype, production stage, disease pressure, climate, ingredient availability, farm scale and market objectives. Recommendations should therefore be based on commercially reproducible responses and transparent diet-level life cycle assessment. Progress towards more sustainable poultry production will require standardized reporting, representative commercial validation and implementation pathways accessible across production scales. Nutritional strategies should ultimately be judged by reproducible net system-level benefits rather than favourable changes in isolated indicators (Figure 3).

## Figures and Tables

**Table 1:** Multidimensional comparison of the efficacy of different organic acid products.

Research Object	Main Components	Addition Dosage	Key Impacts on Intestinal Health	References
Organic acid mixture	Acifed-FS	0.5, 1.0, 1.5 kg/ton	Reduces pH in crop, gizzard and duodenum; elevates *Lactobacillus* abundance and decreases total bacterial count; and improves nutrient digestibility in ileum as well as retention rates of nitrogen, calcium and phosphorus.	Thirumeigmanam et al. [111]
Mixture of organic acids and essential oils	Coated organic acids (sodium butyrate, calcium salts) and essential oils	300 g/ton	Reduces intestinal lesion scores and serum fluorescein isothiocyanate-dextran levels; upregulates the expression of *MUC2*, *CLDN1* and *OCLN* genes; and improves intestinal integrity.	Stefanello et al. [112]
Organic acid mixture	Ammonium formate and calcium propionate	3 g/kg	Increases villus height in all small intestinal segments; reduces *Escherichia coli* counts in intestinal digesta and feed.	Paul et al. [113]
Combination of organic acids and prebiotics	Mannan oligosaccharides (MOS) and butyric acid (BA)	0.1% MOS + 0.3% BA	Enhances antibody titers and cellular immune responses; decreases coliform counts in cecal digesta and excreta while increasing Lactobacillus populations; and upregulates the expression of jejunal *SGLT1*, *PePT1* and *GLUT5* genes.	Raza et al. [114]
Organic acids and essential oils	Mixture of encapsulated organic acids and natural identical essential oils	5 g/kg	Improved growth performance in the final growth stage and some morphological gut traits and reduced to a certain extent Clostridium perfringens count in ileum.	Stamilla et al. [115]
Encapsulated organic acids with essential oils	4% Carvacrol, 4% Thyme, 0.5% Hexanoic, 3.5% Benzoic, and 0.5% Butyric acid	200, 500, 800 mg/kg	Alleviate necrotic enteritis-induced gut impairment and growth depression and modulate cecal microbiota composition.	Phạm et al. [116]

MUC2, Mucin 2; CLDN1, Claudin 1; OCLN, Occludin; SGLT1, sodium-glucose linked transporter 1; PePT1 (PepT1), peptide transporter 1; GLUT5, glucose transporter 5.

**Table 2:** Comparative analysis of different interventions on feed conversion ratio.

Research Subject	Intervention	Feed Form and Pellet Quality	Effect on Feed Conversion Ratio	Reference
Japanese quail genetic selection	Direct selection for reduced 4-week feed conversion ratio over three successive generations	Not reported	Realized heritability of feed conversion ratio reached 0.67; the final generation feed conversion ratio of selected line was 2.13 vs. 2.61 in control line, with a cumulative genetic improvement of 18.4% (6.1% per generation). Selection simultaneously increased body weight and weight gain, and decreased feed intake and residual feed intake.	Varkoohi et al. [137]
Broiler nutritional regulation	Supplementation of compound enzymes (xylanase + phytase)	Not reported	Significantly reduced feed conversion ratio and improved feed efficiency.	Badshah et al. [138]
Broiler nutritional regulation	Dietary supplementation with *Bacillus* spp., *Lactobacillus* spp., or multi-genus mixed probiotics separately	Not reported at the meta-analysis level	All three probiotic categories significantly reduced feed conversion ratio. Standardized effect sizes: Probiotic Mix (−1.32), *Lactobacillus* (−1.22), *Bacillus* (−1.04). Converted to practical metric units, *Lactobacillus* reduced feed conversion ratio by 0.17, Probiotic Mix by 0.14, *Bacillus* by 0.10. Probiotic dosage and strain number had no significant moderating effects.	Opazo et al. [139]
Broiler nutritional regulation	Application of antibiotic growth promoters	Not reported at the meta-analysis level	Overall feed conversion ratio improved by 2.8%.	El-Fateh et al. [140]
Broiler environmental management	Four litter materials (paddy husk, chopped newspaper, coir dust, sand) used for broiler rearing up to 42 days	Not reported	No significant differences in overall feed conversion ratio among all groups *(p* > 0.05). The lowest feed conversion ratio (1.78) was observed in newspaper group, followed by paddy husk (1.79), coir dust (1.82), sand (1.83).	Pagthinathan et al. [141]
Broiler environmental control	Four rearing systems (environmentally controlled house, traditional cage, deep litter, free-range) raised broilers for 42 days	Not reported	Feed conversion ratio differed significantly among all groups (*p* < 0.001). Environmentally controlled house had the lowest feed conversion ratio (1.75), followed by free-range (1.80), deep litter (1.85), and traditional cages with the highest FCR (1.90).	Rauf et al. [142]
Broiler health management	Infection with coccidia (*Eimeria* spp.)	Not reported	Feed conversion ratio increased by 15.4% during the finishing phase and 19.5% over the whole production cycle.	Gickel et al. [143]

Feed form, pellet durability index, hardness and fines were not reported unless otherwise indicated.

**Table 3:** Comprehensive comparison of multi-source alternative proteins in practical production.

Research Subject	Technical Approaches	Research Conclusions	Application Fields	Reference
Black soldier fly larvae	Rearing using organic waste including kitchen residues and agricultural by-products	Efficiently convert waste materials into high-protein biomass; moderate dietary inclusion improves animal growth performance, immunity and intestinal health	Aquaculture (shrimp, fish), poultry (broilers, laying hens, meat ducks), swine	Ling et al. [172]
Fungal protein (*Fusarium venenatum*)	Fermentation combined with CRISPR/Cas9 metabolic engineering modification	Engineered strains achieve an 88.4% higher protein production efficiency, a 32.9% elevated essential amino acid index, alongside reduced global warming potential	Food and feed ingredients	Wu et al. [173]
Single-cell protein (SCP)	Aerobic fermentation with biogas methane or kitchen waste as substrates	Enables nearly 100% energy self-sufficiency; the final product is economically viable and exerts lower environmental impacts than fossil-derived single-cell protein	Animal feed	Dickson et al. [174]
Silkworm pupae meal	Processing of by-products from the silk reeling industry	Contains 49–55% crude protein on a dry matter basis; can fully replace fishmeal and soybean meal without compromising animal growth performance	Poultry, livestock, aquaculture	Chethan et al. [175]
Earthworm meal	Artificial breeding and post-harvest processing	Recommended maximum inclusion rate of 15% in broiler diets; suitable for incorporation at 25–30% in aquafeeds	Poultry, aquaculture	Arshad et al. [176]
Plant protein (pea seeds + rapeseed meal)	Gradient partial replacement of genetically modified soybean meal at 5.0%, 10.0%, 15.0% and 17.5% in two-stage isoenergetic and isonitrogenous grow-finish pig diets; determination of pork physicochemical traits, intramuscular fatty acid composition and lipid health indices	Dietary pea inclusion lowered muscle saturated fatty acids, boosted polyunsaturated fatty acid proportion, reduced atherogenicity index, thrombogenicity index and saturated/polyunsaturated fatty acid ratio while elevating Hypocholesterolemic fatty acids/Hypercholesterolemic fatty acids ratio to improve pork nutritional value	Swine	Sońta et al. [177]
Blended plant protein blends	Combination of corn gluten meal, sunflower meal and DDGS to substitute soybean meal in broiler feeds	Broilers fed the replacement diet containing 21% crude protein exhibit superior European Production Efficiency Factor and economic profitability	Broilers	El-Deek et al. [178]
*Chlorella vulgaris* biomass	Partial replacement of soybean meal with 10%, 15% or 20% *C. vulgaris* in diets offered to male Ross 308 broilers for 40 days	A 10% inclusion maintained growth performance, whereas 15% and 20% reduced body weight gain and feed intake. Higher inclusion improved several breast-meat quality and n-3 fatty-acid traits but increased ileal digesta viscosity, indicating a dose-dependent practical limit	Broilers	Boskovic Cabrol et al. [179]

**Table 4:** Comparative analysis of land use changes driven by soybean cultivation.

Research Object	Study Period	Dominant Land Use Transition Pathways	Carbon Emission Impacts	Key Findings	Reference
Seven tropical countries (including Brazil and Argentina)	2000–2011	Deforestation driven by the production of beef, soybeans, palm oil and other agricultural commodities	Responsible for 40% of tropical deforestation and associated carbon losses	Over one-third of embodied carbon emissions are embedded in export commodities, with growing influences exerted by global commodity markets	Henders et al. [213]
Cerrado biome, Brazil	2003–2013	Conversion of native forests to croplands	Annual carbon emissions of 16.28 Tg C; forest-to-cropland conversion accounts for 29% of total emissions	Carbon emissions from forest loss in the Matopiba agricultural frontier rose to 45% during 2010–2013	Noojipady et al. [214]
Whole territory of Brazil	1940–1995	Conversion of native forests and natural grasslands to croplands and cattle ranches	Net carbon emission of 17.2 ± 9.0 Pg C	72% of total carbon emissions stem from the Atlantic Forest and Cerrado biomes rather than the Amazon rainforest	Leite et al. [215]
Gran Chaco region, Argentina	1985–2013	Sequential conversion: native forests converted to ranches, followed by ranch-to-cropland transformation	Cumulative carbon emissions of 824 Tg C; forest-to-ranch conversion contributes 68% of total emissions	Carbon emissions from deforestation in tropical dry forests are comparable to those generated at major humid Amazon deforestation frontiers	Baumann et al. [216]
Mato Grosso, Brazilian Amazon	2001–2004	Direct clearance of native forests for soybean cultivation	Not reported	Direct deforestation area exceeded 540,000 hectares, exhibiting a strong positive correlation with soybean prices (R^2^ = 0.72)	Morton et al. [217]
Mato Grosso, Brazilian Amazon	2004–2014	Direct and indirect deforestation at the farm scale	Not reported	Indirect deforestation accounted for more than half of total forest loss after the implementation of the Soy Moratorium	Gollnow et al. [218]
Southern Brazilian Amazon	2001–2005	Wildfires triggered by deforestation and pasture management practices	Annual carbon emissions of 67 Tg C from deforestation fires	Large-scale vegetation clearances over 100 hectares contribute 67% of total fire-related carbon emissions	DeFries et al. [219]

**Table 5:** Minimum reporting protocol * for life cycle assessment of poultry nutritional strategies.

Protocol Element	Minimum Requirement
Goal and comparator	State the decision question, production system, baseline diet and counterfactual. Compare complete diets or feeding programmes when animal performance differs.
Functional unit	Use 1 kg live-weight gain or carcass weight for meat poultry and 1 kg marketable egg mass for laying hens. Feed mass may be reported only as an auxiliary unit for upstream feed burdens.
System boundary	Include crop and ingredient production, relevant land-use change, additive and crystalline amino acid manufacture, packaging, transport, milling and pelleting, farm energy, animal performance, mortality and manure management.
Biological performance	Use treatment-specific feed intake, body-weight gain or egg mass, mortality and production duration. Pellet quality should be reported when it may affect intake or FCR.
Allocation	Declare the allocation method used for crops, oilseed meals, fermentation coproducts, insects, microalgae, and agro-industrial residues and test plausible alternatives.
Impact categories	Report climate change together with acidification, eutrophication, land use, water use and cumulative energy demand where data permit.
Additive-related burdens	Include substrates, fermentation or extraction, heat, aeration, separation, drying, encapsulation, carriers, packaging, transport and shelf-life losses.
Data quality	Report geographical origin, reference year, technological representativeness and the proportion of primary versus database-derived data.
Uncertainty	Conduct sensitivity analyses for ingredient origin, energy mix, allocation, land-use change, additive stability and biological response.
Reporting	Provide a system-boundary diagram, inventory sources, assumptions, exclusions and results disaggregated by life cycle stage.

The protocol was developed with reference to ISO 14040, ISO 14044 and the European Commission Environmental Footprint method [264,265,266] and was adapted to the evaluation of poultry nutritional strategies.

## Data Availability

No new data were created or analyzed in this study. Data sharing is not applicable to this article.

## Abbreviations

The following abbreviations are used in this manuscript:

AALP	Amino Acid-Balanced Low-Protein
AGPs	Antibiotic Growth Promoters
AMR	Antimicrobial Resistance
CP	Crude Protein
FCR	Feed Conversion Ratio
GHG	Greenhouse Gas
LCA	Life Cycle Assessment
N_2_O	Nitrous Oxide
NH_3_	Ammonia

## References

[1] Tian S., Guo J., Wang X., Zhang J., He M., Ma Y., Wang Y. (2026). Opportunities, constraints and transforming sustainable poultry production in Ethiopia: A review (2010–2025). J. Sci. Res. Rep..

[2] Kang H.-Y., Hwang Y.W., Lee J.H., Cho S.J., Jeon Y.J., Kim N.S., Kim J. (2025). Evaluating the greenhouse gas emissions footprint of chicken meat production in South Korea: A life cycle perspective. Food Bioprod. Process..

[3] Cheng Z., Jia Y., Bai Y., Zhang T., Ren K., Zhou X., Zhai Y., Shen X., Hong J. (2022). Intensifying the environmental performance of chicken meat production in China: From perspective of life cycle assessment. J. Clean. Prod..

[4] de Verdal H., Mignon-Grasteau S., Bastianelli D., Même N., Le Bihan-Duval E., Narcy A. (2012). Reducing the environmental impact of poultry breeding by genetic selection. J. Anim. Sci..

[5] van Hal O., Weijenberg A., de Boer I.J.M., van Zanten H.H.E. (2019). Accounting for feed-food competition in environmental impact assessment: Towards a resource efficient food-system. J. Clean. Prod..

[6] Tilman D., Fargione J., Wolff B., D’Antonio C., Dobson A., Howarth R., Schindler D., Schlesinger W.H., Simberloff D., Swackhamer D. (2001). Forecasting agriculturally driven global environmental change. Science.

[7] Chandrakar P., Bhupender, Yadav R.S., Garg D., Ujjwal P., Kumar D., Gupta A. (2026). Modern livestock production management strategies for resource-efficient animal agriculture: A review. Arch. Curr. Res. Int..

[8] Salami S.A., Taylor-Pickard J., Ross S.A., Moran C.A. (2024). A meta-analysis of the effects of dietary yeast mannan-rich fraction on broiler performance and the implication for greenhouse gas emissions from chicken production. Animals.

[9] Pin Viso N., Redondo E.A., Redondo L.M., Díaz Carrasco J., Farber M., Fernández Miyakawa M. (2025). Could tannins be the right dietary alternative for replacing antibiotics as growth promoters in broiler chicken production? A comprehensive microbiota shift assessment in a commercial farm. Poult. Sci..

[10] Salinas-Chavira J., Barrios-García H. (2024). Essential oils, chemical compounds, and their effects on the gut microorganisms and broiler chicken production: Review. Agriculture.

[11] Soren S., Mandal G.P., Mondal S., Pradhan S., Mukherjee J., Banerjee D., Pakhira M.C., Amla, Mondal A., Nsereko V. (2024). Efficacy of *Saccharomyces cerevisiae* fermentation product and probiotic supplementation on growth performance, gut microflora and immunity of broiler chickens. Animals.

[12] Wang Z.R., Qiao S., Lu W.Q., Li D.F. (2005). Effects of enzyme supplementation on performance, nutrient digestibility, gastrointestinal morphology, and volatile fatty acid profiles in the hindgut of broilers fed wheat-based diets. Poult. Sci..

[13] Tang Z., Wen C., Li P., Wang T., Zhou Y. (2014). Effect of zinc-bearing zeolite clinoptilolite on growth performance, nutrient retention, digestive enzyme activities, and intestinal function of broiler chickens. Biol. Trace Elem. Res..

[14] Isusi S., Usero-Alonso G., Murillo J.A., Gonzalez-Guijarro A.B., Muñoz A., Ramis G. (2025). Influence of nutritional strategies on performance, gut barrier function and microbiota composition in weaned piglets. Animals.

[15] Ji M., Liu J., Wang Q., Gong T., Zhang J., Li M., Guo X., Yang Y., Li B. (2026). Optimization of solid-state fermentation process of diets and its effects on growth performance and intestinal function in growing pigs. Anim. Microbiome.

[16] Anderson R.C., Cookson A.L., McNabb W.C., Park Z., McCann M.J., Kelly W.J., Roy N.C. (2010). *Lactobacillus plantarum* MB452 enhances the function of the intestinal barrier by increasing the expression levels of genes involved in tight junction formation. BMC Microbiol..

[17] Cani P.D., Possemiers S., Van de Wiele T., Guiot Y., Everard A., Rottier O., Geurts L., Naslain D., Neyrinck A., Lambert D.M. (2009). Changes in gut microbiota control inflammation in obese mice through a mechanism involving GLP-2-driven improvement of gut permeability. Gut.

[18] Wu Y., Zhen W., Geng Y., Wang Z., Guo Y. (2019). Pretreatment with probiotic *Enterococcus faecium NCIMB 11181* ameliorates necrotic enteritis-induced intestinal barrier injury in broiler chickens. Sci. Rep..

[19] Nawab A., Dao T.H., Chrystal P.V., Cadogan D., Wilkinson S., Kim E., Crowley T., Barekatain R., Moss A.F. (2025). Evaluation of precision feeding to enhance broiler growth performance. Animals.

[20] Kim J.D., Saremi B., Siegert W., Sulabo R.C., Tatekura S. (2026). Steering nitrogen emission from poultry production through precision nutrition. Poult. Sci..

[21] Remus A., Hauschild L., Létourneau-Montminy M.-P., Corrent E., Pomar C. (2020). The ideal protein profile for late-finishing pigs in precision feeding systems: Threonine. Anim. Feed Sci. Technol..

[22] Narote A.D., Casson A., Giovenzana V., Pampuri A., Tugnolo A., Beghi R., Guidetti R. (2025). Integrating environmental, nutritional, and economic dimensions in food choices: A case study on legume vs. meat-based burger patties. J. Agric. Food Res..

[23] Springmann M., Clark M., Mason-D’Croz D., Wiebe K., Bodirsky B.L., Lassaletta L., de Vries W., Vermeulen S.J., Herrero M., Carlson K.M. (2018). Options for keeping the food system within environmental limits. Nature.

[24] Cordero P., Ramírez-Toloza G., Dufflocq P., Herrera-Alcaíno S., Guzmán-Pino S.A. (2025). Reduced dietary protein and essential amino acids impair growth performance and increase lysine sensitivity in broiler chickens. Animals.

[25] Attia Y.A., Bovera F., Wang J., Al-Harthi M.A., Kim W.K. (2020). Multiple amino acid supplementations to low-protein diets: Effect on performance, carcass yield, meat quality and nitrogen excretion of finishing broilers under hot climate conditions. Animals.

[26] de Rauglaudre T., Méda B., Fontaine S., Lambert W., Fournel S., Létourneau-Montminy M.-P. (2023). Meta-analysis of the effect of low-protein diets on the growth performance, nitrogen excretion, and fat deposition in broilers. Front. Anim. Sci..

[27] Selle P.H., Dorigam J.C.d.P., Lemme A., Chrystal P.V., Liu S.Y. (2020). Synthetic and crystalline amino acids: Alternatives to soybean meal in chicken-meat production. Animals.

[28] Mansilla W.D., Saraswathy S., García-Ruiz A.I. (2023). Dietary protein reduction with stepwise addition of crystalline amino acids and the effect of considering a minimum glycine-serine content in broiler diets. Poult. Sci..

[29] Bovera F., Loponte R., Pero M.E., Cutrignelli M.I., Calabrò S., Musco N., Vassalotti G., Panettieri V., Lombardi P., Piccolo G. (2018). Laying performance, blood profiles, nutrient digestibility and inner organs traits of hens fed an insect meal from *Hermetia illucens* larvae. Res. Vet. Sci..

[30] Li Z., Cao Y., Liu M., Yuan J., Li J., Huo W., Huang M., Kang S., Sui S., Ma X. (2026). Feasibility evaluation for cockroach from *Periplaneta americana* as a new protein feed ingredient in pigs. Anim. Feed Sci. Technol..

[31] Hartinger K., Greinix J., Thaler N., Ebbing M.A., Yacoubi N., Schedle K., Gierus M. (2021). Effect of graded substitution of soybean meal by *Hermetia illucens* larvae meal on animal performance, apparent ileal digestibility, gut histology and microbial metabolites of broilers. Animals.

[32] Srinual O., Kanmanee C., Srinual P., Chaiyaso T., Yachai M., Tapingkae T., Tapingkae W. (2024). Innovation and utilization of functional feed additives from maize by-products in broiler chickens. Animals.

[33] Younis M., Abdo S.G., Abu Elmakarem M.A., Mustafa F.E.-Z.A., Fawaz M.A. (2025). Evaluating dried pomegranate peel as a functional feed additive: Effects on growth, carcass traits, and gut health in broilers. Trop. Anim. Health Prod..

[34] Ogino A., Oishi K., Setoguchi A., Osada T. (2021). Life cycle assessment of sustainable broiler production systems: Effects of low-protein diet and litter incineration. Agriculture.

[35] Hickmann F., Andretta I., Létourneau-Montminy M.-P., Remus A., Galli G.M., Vittori J., Kipper M. (2021). Β-Mannanase supplementation as an eco-friendly feed strategy to reduce the environmental impacts of pig and poultry feeding programs. Front. Vet. Sci..

[36] Cappelaere L., Le Cour Grandmaison J., Bas Martín N., Lambert W. (2021). Amino acid supplementation to reduce environmental impacts of broiler and pig production: A review. Front. Vet. Sci..

[37] Mosnier E., van der Werf H., Boissy J., Dourmad J.-Y. (2011). Evaluation of the environmental implications of the incorporation of feed-use amino acids in the manufacturing of pig and broiler feeds using life cycle assessment. Animal.

[38] Kebreab E., Liedke A., Caro D., Deimling S., Binder M.D., Finkbeiner M. (2016). Environmental impact of using specialty feed ingredients in swine and poultry production: A life cycle assessment. J. Anim. Sci..

[39] Leinonen I., Williams A. (2015). Effects of dietary protease on nitrogen emissions from broiler production: A holistic comparison using Life Cycle Assessment. J. Sci. Food Agric..

[40] Idan F., Nortey T.N., Paulk C.B., Beyer R., Stark C.R. (2020). Evaluating the effect of feeding starters crumbles on the overall performance of broilers raised for 42 days. J. Appl. Poult. Res..

[41] Paul S.S., Rama Rao S.V., Chatterjee R.N., Raju M.V.L.N., Prakash B., Yadav S.P., Kannan A., Reddy G.N., Kumar V., Kumar P.S.P. (2025). Evaluation of phytase super dosing and its combinations with other additives as an alternative to antibiotic growth promoters in broiler chickens reared in a deep litter system. Front. Anim. Sci..

[42] Đurić Jarić M., Gottstein Ž., Vince S., Žura Žaja I., Brus M., Đuričić D., Samardžija M., Valpotić H. (2025). Effect of standardized ginger (*Zingiber officinale* Roscoe) extract on gut morphology, microbiota composition, and growth performance in broiler chickens. Agriculture.

[43] Ghareeb K., König K., Awad W.A., Zebeli Q., Böhm J. (2015). The impact of a microbial feed supplement on small intestine integrity and oxidative stress biomarker in broiler chickens. Avian Biol. Res..

[44] Xu H., Gong L., Zhang X., Li Z., Fu J., Lv Z., Guo Y. (2024). Effects of tannic acid on growth performance, intestinal health, and tolerance in broiler chickens. Poult. Sci..

[45] Peng H., Song X., Chen J., Xiong X., Yang L., Yu C., Qiu M., Zhang Z., Hu C., Zhu S. (2024). Soybean bioactive peptide supplementation improves gut health and metabolism in broiler chickens. Poult. Sci..

[46] Wang D., Hu F., Liu H., She R., Tian J. (2025). Effects of chicken hemoglobin antimicrobial peptides on intestinal mucosal immunity under chronic heat stress and vaccination responses in broilers. Front. Anim. Sci..

[47] Haldar S., Dhara A.K., Sengupta I., Paul S., Arora S.S., Kumar R., Pal A., Kulkarni V. (2025). A novel subtilisin-producing *Bacillus velezensis* strain (BV-OLS1101) improves growth and mucosal immunity in broiler chickens under Clostridium perfringens challenge. Poult. Sci..

[48] Perricone V., Comi M., Irshad N., Lecchi C., Dadi Y., Aidos L., Cremonesi P., Castiglioni B., Biscarini F., Savoini G. (2025). Impact of yeast-derived nucleotides supplementation in drinking water on production performance and gut health indicators in broiler chickens. Poult. Sci..

[49] Yu X., Dai Z., Cao G., Cui Z., Zhang R., Xu Y., Wu Y., Yang C. (2023). Protective effects of *Bacillus licheniformis* on growth performance, gut barrier functions, immunity and serum metabolome in lipopolysaccharide-challenged weaned piglets. Front. Immunol..

[50] Wu H., Zhao D., Yu X., Chai H., Liu H., Peng W., Xu L., Hou G. (2026). N-carbamylglutamate promotes growth and immunity in danzhou chickens via gut microbiota-metabolite interactions involving sphingolipid and mTOR pathways. Microorganisms.

[51] Wu H., Chai H., Yu X., Zhao D., Liu H., Peng W., Ji F., Xu L., Hou G. (2026). Alpinia katsumadai and Wurfbainia vera extracts modulate antioxidant function and intestinal morphology in danzhou chickens via gut microbiota–metabolite interactions involving hydroxyoctadecadienoic acid metabolism and bacteroidota remodeling. Microorganisms.

[52] Arpaia N., Campbell C., Fan X., Dikiy S., van der Veeken J., deRoos P., Liu H., Cross J.R., Pfeffer K., Coffer P.J. (2013). Metabolites produced by commensal bacteria promote peripheral regulatory T-cell generation. Nature.

[53] Jain U., Lai C.W., Xiong S., Goodwin V.M., Lu Q., Muegge B.D., Christophi G.P., VanDussen K.L., Cummings B.P., Young E. (2018). Temporal regulation of the bacterial metabolite deoxycholate during colonic repair is critical for crypt regeneration. Cell Host Microbe.

[54] Neis E.P.J.G., Dejong C.H.C., Rensen S.S. (2015). The role of microbial amino acid metabolism in host metabolism. Nutrients.

[55] Zhu J., Wang L., Xu H., Ma Z., Tao J. (2024). Fagopyrum cymosum alleviates DSS-induced colitis via ameliorating intestinal barrier function and remolding gut microbiota. J. Funct. Foods.

[56] Dai Y., Lin Z., Zhang X., Wang Y., Sheng Y., Gao R., Geng Y., Xue Y., Ren Y. (2025). Probiotic Lacticaseibacillus paracasei E10 ameliorates dextran sulfate sodium-induced colitis by enhancing the intestinal barrier and modulating microbiota. Foods.

[57] Gao Y., Long M., Xu M., Yang T., Li J., Liu M., Ma J., Du Y., Xu Q. (2025). Alginate oligosaccharide attenuates lipopolysaccharide-induced intestinal barrier dysfunction in Balb/c mice: Mechanistic insights. J. Agric. Food Chem..

[58] Bahlouli W., Breton J., Lelouard M., L’Huillier C., Tirelle P., Salameh E., Amamou A., Atmani K., Goichon A., Bôle-Feysot C. (2020). Stress-induced intestinal barrier dysfunction is exacerbated during diet-induced obesity. J. Nutr. Biochem..

[59] Jo H.E., Kwon M.-S., Whon T.W., Kim D.W., Yun M., Lee J.-E., Shin M.-Y., Kim S.-H., Choi H.-J. (2021). Alteration of gut microbiota after antibiotic exposure in finishing swine. Front. Microbiol..

[60] Gao P., Hou Q., Kwok L.-Y., Huo D., Feng S., Zhang H. (2017). Effect of feeding *Lactobacillus plantarum* P-8 on the faecal microbiota of broiler chickens exposed to lincomycin. Sci. Bull..

[61] Xu Y., Huang Y., Wei S., Tian J., Huang Y., Nie Q., Zhang D. (2025). Changes in gut microbiota affect DNA methylation levels and development of chicken muscle tissue. Poult. Sci..

[62] El Fatihi I., Khodja S., Bakr M.A., Rahim M.F., Li S., Nawaz S., Ijaz F., Jin C., Li J. (2026). Cadmium chloride administered through drinking water disrupts the gut-liver axis through intestinal barrier failure, endoplasmic reticulum stress, and PIEZO1 signaling in broiler chickens. Poult. Sci..

[63] Zou H., Ali W., Deng K., Chen Y., Sun J., Wang T., Liu Z. (2024). The protective effect of luteolin on cadmium induced liver intestinal toxicity in chicken by Gut-liver axis regulation. Poult. Sci..

[64] Carpino G., Del Ben M., Pastori D., Carnevale R., Baratta F., Overi D., Francis H., Cardinale V., Onori P., Safarikia S. (2019). Increased liver localization of lipopolysaccharides in human and experimental NAFLD. Hepatology.

[65] Zhang C., Song Y., Yuan M., Chen L., Zhang Q., Hu J., Meng Y., Li S., Zheng G., Qiu Z. (2023). Ellagitannins-derived intestinal microbial metabolite urolithin A ameliorates fructose-driven hepatosteatosis by suppressing hepatic lipid metabolic reprogramming and inducing lipophagy. J. Agric. Food Chem..

[66] Qiao Y., Zhang Z., Zhai Y., Yan X., Zhou W., Liu H., Guan L., Peng L. (2022). Apigenin alleviates obesity-associated metabolic syndrome by regulating the composition of the gut microbiome. Front. Microbiol..

[67] Kim D.-h., Sim Y., Hwang J.-h., Kwun I.-S., Lim J.-H., Kim J., Kim J.-I., Baek M.-C., Akbar M., Seo W. (2021). Ellagic acid prevents binge alcohol-induced leaky gut and liver injury through inhibiting gut dysbiosis and oxidative stress. Antioxidants.

[68] Chang C.J., Lin C.S., Lu C.C., Martel J., Ko Y.F., Ojcius D.M., Tseng S.F., Wu T.R., Chen Y.Y.M., Young J.D. (2015). Ganoderma lucidum reduces obesity in mice by modulating the composition of the gut microbiota. Nat. Commun..

[69] Awad W.A., Ghareeb K., Abdel-Raheem S.M., Böhm J. (2008). Effects of dietary inclusion of probiotic and synbiotic on growth performance, organ weights, and intestinal histomorphology of broiler chickens. Poult. Sci..

[70] Tukaram N.M., Biswas A., Deo C., Laxman A.J., Monika M., Tiwari A.K. (2022). Effects of paraprobiotic as replacements for antibiotic on performance, immunity, gut health and carcass characteristics in broiler chickens. Sci. Rep..

[71] Gueimonde M., Sánchez B., de Los Reyes-Gavilán C.G., Margollés A. (2013). Antibiotic resistance in probiotic bacteria. Front. Microbiol..

[72] Su G.L., Ko C.W., Berčík P., Falck–Ytter Y., Sultan S., Weizman A.V., Morgan R.L. (2020). AGA clinical practice guidelines on the role of probiotics in the management of gastrointestinal disorders. Gastroenterology.

[73] Dalloul R.A., Lillehoj H.S., Shellem T.A., Doerr J.A. (2003). Enhanced mucosal immunity against *Eimeria acervulina* in broilers fed a Lactobacillus-based probiotic. Poult. Sci..

[74] Rasaei D., Hosseinian S.A., Asasi K., Shekarforoush S.S., Khodakaram-Tafti A. (2023). The beneficial effects of spraying of probiotic Bacillus and Lactobacillus bacteria on broiler chickens experimentally infected with avian influenza virus H9N2. Poult. Sci..

[75] Tu Z., Setlow P., Brul S., Kramer G. (2021). Molecular physiological characterization of a high heat resistant spore forming *Bacillus subtilis* food isolate. Microorganisms.

[76] Sella S.R.B.R., Vandenberghe L.P.S., Soccol C.R. (2014). Life cycle and spore resistance of spore-forming *Bacillus* atrophaeus. Microbiol. Res..

[77] Li Z., Liu W., Wu H., Peng S., Zhao M., Lin F., Zhao L. (2025). Effects of *Lactiplantibacillus-plantarum*-ZG7-Fermented feed on laying-hen productivity and intestinal health. Fermentation.

[78] Humam A.M., Loh T.C., Foo H.L., Samsudin A.A., Mustapha N.M., Zulkifli I., Izuddin W.I. (2019). Effects of feeding different postbiotics produced by *Lactobacillus plantarum* on growth performance, carcass yield, intestinal morphology, gut microbiota composition, immune status, and growth gene expression in broilers under heat stress. Animals.

[79] Zaman S.A., Sarbini S.R. (2015). The potential of resistant starch as a prebiotic. Crit. Rev. Biotechnol..

[80] Wiese M., van der Wurff M., Ouwens A., van Leijden B., Verheij E.R., Heerikhuisen M., van der Vossen J.M.B.M. (2024). Modeling the effects of prebiotic interventions on luminal and mucosa-associated gut microbiota without and with *Clostridium difficile* challenge in vitro. Front. Nutr..

[81] Kim G., Seo Y.-J., Kim C.H., Paik I.K. (2010). Effect of dietary prebiotic supplementation on the performance, intestinal microflora, and immune response of broilers. Poult. Sci..

[82] Jazi V., Foroozandeh A.D., Toghyani M., Toghyani M., Dastar B., Koochaksaraie R.R., Toghyani M. (2018). Effects of *Pediococcus acidilactici*, mannan-oligosaccharide, butyric acid and their combination on growth performance and intestinal health in young broiler chickens challenged with *Salmonella* Typhimurium. Poult. Sci..

[83] Macfarlane S., Cleary S., Bahrami B., Reynolds N.J., Macfarlane G.T. (2013). Synbiotic consumption changes the metabolism and composition of the gut microbiota in older people and modifies inflammatory processes: A randomised, double-blind, placebo-controlled crossover study. Aliment. Pharmacol. Ther..

[84] Deshan N., Huang Y., Zhou Y., Zhao H., Nie W., Zheng Y., Huang X. (2024). Evaluation of the immunomodulatory effects of a novel synbiotic made of combined use of probiotic-prebiotic-Chinese traditional herbs. J. Agric. Food Res..

[85] Azagra-Boronat I., Massot-Cladera M., Knipping K., Garssen J., Ben Amor K., Knol J., Franch À., Castell M., Rodríguez-Lagunas M.J., Pérez-Cano F.J. (2020). Strain-specific probiotic properties of bifidobacteria and lactobacilli for the prevention of diarrhea caused by rotavirus in a preclinical model. Nutrients.

[86] Ramos C.L., Thorsen L., Schwan R.F., Jespersen L. (2013). Strain-specific probiotics properties of *Lactobacillus fermentum*, *Lactobacillus plantarum* and *Lactobacillus brevis* isolates from Brazilian food products. Food Microbiol..

[87] Soto L.P., Frizzo L.S., Avataneo E., Zbrun M.V., Bertozzi E., Sequeira G., Signorini M., Rosmini M.R. (2011). Design of macrocapsules to improve bacterial viability and supplementation with a probiotic for young calves. Anim. Feed Sci. Technol..

[88] Dong H.T., Nguyen V.V., Le H.D., Sangsuriya P., Jitrakorn S., Saksmerprome V., Senapin S., Rodkhum C. (2015). Naturally concurrent infections of bacterial and viral pathogens in disease outbreaks in cultured Nile tilapia (*Oreochromis niloticus*) farms. Aquaculture.

[89] Zhang H., Wu Z.H., Sun X.M., Sun C., Chang J., Yuan L. (2026). Gender-specific and dose-dependent responses to L-se-methylselenocysteine are mediated by the gut microbiota-metabolite axis: Implications for intestinal homeostasis and safe clinical application. Front. Nutr..

[90] Jiang J.W., Ren Z.G., Lu H.F., Zhang H., Li A., Cui G.Y., Jia J.J., Xie H.Y., Chen X.H., He Y. (2018). Optimal immunosuppressor induces stable gut microbiota after liver transplantation. World J. Gastroenterol..

[91] Murugesan G.R., Syed B., Haldar S., Pender C. (2015). Phytogenic feed additives as an alternative to antibiotic growth promoters in broiler chickens. Front. Vet. Sci..

[92] Basiouni S., Tellez-Isaias G., Latorre J.D., Graham B.D., Petrone-Garcia V.M., El-Seedi H.R., Yalçın S., El-Wahab A.A., Visscher C., May-Simera H.L. (2023). Anti-inflammatory and antioxidative phytogenic substances against secret killers in poultry: Current status and prospects. Vet. Sci..

[93] Varmuzova K., Elsheimer Matulova M., Gerzova L., Cejkova D., Gardan-Salmon D., Panhéleux M., Robert F., Sisak F., Havlickova H., Rychlik I. (2015). *Curcuma* and *Scutellaria* plant extracts protect chickens against inflammation and *Salmonella* Enteritidis infection. Poult. Sci..

[94] Abd El-Ghany W.A., Soyadı Y.A.Y. (2020). Phytobiotics in poultry industry as growth promoters, antimicrobials and immunomodulators—A review. J. World’s Poult. Res..

[95] Nantapo C.W.T., Marume U. (2025). Strategic technologies to improve phytogenic feed additive efficacy in pigs and poultry. Anim. Nutr..

[96] Rafeeq M., Bilal R.M., Batool F., Yameen K., Farag M.R., Madkour M., Elnesr S.S., El-Shall N.A., Dhama K., Alagawany M. (2023). Application of herbs and their derivatives in broiler chickens: A review. World’s Poult. Sci. J..

[97] Rafeeq M., Bilal R.M., Alagawany M., Batool F., Yameen K., Farag M.R., Ali S., Elnesr S.S., El-Shall N.A. (2022). The use of some herbal plants as effective alternatives to antibiotic growth enhancers in poultry nutrition. World’s Poult. Sci. J..

[98] Gil-Sánchez I., Cueva C., Sanz-Buenhombre M., Guadarrama A., Moreno-Arribas M.V., Bartolomé B. (2017). Dynamic gastrointestinal digestion of grape pomace extracts: Bioaccessible phenolic metabolites and impact on human gut microbiota. J. Food Compos. Anal..

[99] Freire F., Adorno M.Â.T., Sakamoto I.K., Antoniassi R., Chaves A.C.S.D., Santos K.M.O.D., Sivieri K. (2017). Impact of multi-functional fermented goat milk beverage on gut microbiota in a dynamic colon model. Food Res. Int..

[100] Hervert-Hernández D., Pintado C., Rotger R., Goñi I. (2009). Stimulatory role of grape pomace polyphenols on *Lactobacillus acidophilus* growth. Int. J. Food Microbiol..

[101] Liu Z., Chen Z., Guo H., He D., Zhao H., Wang Z., Zhang W., Liao L., Zhang C., Ni L. (2016). The modulatory effect of infusions of green tea, oolong tea, and black tea on gut microbiota in high-fat-induced obese mice. Food Funct..

[102] Wang M., Mao Y., Wang B., Wang S., Han L., Ying L., Li Y. (2020). Quercetin improving lipid metabolism by regulating lipid metabolism pathway of ileum mucosa in broilers. Oxid. Med. Cell. Longev..

[103] Xiao P., Li Q., Wang L., Sun G., Hou H., Han X., Feng J., Min Y. (2025). The combined supplementation of quercetin and *Ligilactobacillus salivarius* improves production performance, lipid metabolism, and ileal health in late-phase laying hens. Poult. Sci..

[104] Ru M., Wang W., Zhai Z., Wang R., Li Y., Jiang L., Kothari D., Niu K.-M., Wu X. (2022). Nicotinamide mononucleotide supplementation protects the intestinal function in aging mice and d-galactose induced senescent cells. Food Funct..

[105] Shamoto K., Yamauchi K. (2000). Recovery responses of chick intestinal villus morphology to different refeeding procedures. Poult. Sci..

[106] Komijani M., Rostami H., Parastouei K., Fathi M. (2025). Fabrication, characterization, and effectiveness of Rosmarinus officinalis L. essential oil nanoencapsulated by chitosan against *Aspergillus flavus* and *Aspergillus parasiticus* in vitro. LWT.

[107] Adil S., Banday M.T., Hussain S.A., Wani M.A., Al-Olayan E., Patra A.K., Rasool S., Gani A., Sheikh I.U., Khan A.A. (2024). Impact of nanoencapsulated rosemary essential oil as a novel feed additive on growth performance, nutrient utilization, carcass traits, meat quality and gene expression of broiler chicken. Foods.

[108] de Souza B.V.C., de Morais Sousa M., Sattler J.A.G., Santana A.C.S.G.V., de Carvalho R.B.F., de Sousa Lima Neto J., de Matos Borges F., Neri Numa I.A., Ribeiro A.B., Nunes L.C.C. (2022). Nanoencapsulation and bioaccessibility of polyphenols of aqueous extracts from *Bauhinia forficata* link. Food Chem. Mol. Sci..

[109] Jiang X., Zou Z., Zeng Y.J., Yuan H., Feng X., Wang Y. (2026). Tannic acid supplementation exerts biphasic effects on growth performance, immune function, and gut microbiota in Pekin duck. Poult. Sci..

[110] Young J.F., Duthie S.J., Milne L., Christensen L.P., Duthie G.G., Bestwick C.S. (2007). Biphasic effect of falcarinol on CaCo-2 cell proliferation, DNA damage, and apoptosis. J. Agric. Food Chem..

[111] Thirumeigmanam D., Swain R.K., Mohanty S., Pati P.K. (2006). Effect of dietary supplementation of organic acids on performance of broiler chicken. Indian J. Anim. Nutr..

[112] Stefanello C., da Rosa D.P., Dalmoro Y.K., Segatto A.L.A., Vieira M.S., de Moraes M.L., Santín E. (2020). Protected blend of organic acids and essential oils improves growth performance, nutrient digestibility, and intestinal health of broiler chickens undergoing an intestinal challenge. Front. Vet. Sci..

[113] Paul S.K., Halder G., Mondal M.K., Samanta G. (2007). Effect of organic acid salt on the performance and gut health of broiler chicken. J. Poult. Sci..

[114] Raza M., Biswas A., Mandal A.B. (2019). Immune response, expression of nutrient transporter genes and gut health status of broiler chicken fed diet supplemented with prebiotic and organic acid. Indian J. Poult. Sci..

[115] Stamilla A., Messina A., Sallemi S., Condorelli L., Antoci F., Puleio R., Loria G.R., Cascone G., Lanza M. (2020). Effects of Microencapsulated Blends of Organics Acids (OA) and Essential Oils (EO) as a Feed Additive for Broiler Chicken. A Focus on Growth Performance, Gut Morphology and Microbiology. Animals.

[116] Pham V.H., Abbas W., Huang J., He Q., Zhen W., Guo Y., Wang Z. (2021). Effect of blending encapsulated essential oils and organic acids as an antibiotic growth promoter alternative on growth performance and intestinal health in broilers with necrotic enteritis. Poult. Sci..

[117] Wolever T.M., Spadafora P., Eshuis H. (1991). Interaction between colonic acetate and propionate in humans. Am. J. Clin. Nutr..

[118] Chang P.V., Hao L., Offermanns S., Medzhitov R. (2014). The microbial metabolite butyrate regulates intestinal macrophage function via histone deacetylase inhibition. Proc. Natl. Acad. Sci. USA.

[119] Zhao X.-H., Yang L. (2009). Resistant starch prepared from high-amylose maize starch with citric acid hydrolysis and its simulated fermentation in vitro. Eur. Food Res. Technol..

[120] Zhang Y., Hu Q., Zeng X., Yin L., Tang Y., Wang Q., Huang J., Li J., Yang H. (2026). Intestinal organoid screen reveals that *Bacillus velezensis PGM541* promotes epithelial proliferation via its metabolite butyric acid. Microbiome.

[121] Dittoe D.K., Ricke S.C., Kiess A.S. (2018). Organic acids and potential for modifying the avian gastrointestinal tract and reducing pathogens and disease. Front. Vet. Sci..

[122] Kuang Y., Wang Y., Zhang Y., Song Y., Zhang X., Lin Y., Che L., Xu S., Wu D., Xue B. (2015). Effects of dietary combinations of organic acids and medium chain fatty acids as a replacement of zinc oxide on growth, digestibility and immunity of weaned pigs. Anim. Feed Sci. Technol..

[123] Thananimint S., Pahumunto N., Teanpaisan R. (2022). Characterization of short chain fatty acids produced by selected potential probiotic lactobacillus strains. Biomolecules.

[124] Shurson G.C., Salzer T.M., Koehler D., Whitney M. (2010). Effect of metal specific amino acid complexes and inorganic trace minerals on vitamin stability in premixes. Anim. Feed Sci. Technol..

[125] Jahanian R. (2009). Immunological responses as affected by dietary protein and arginine concentrations in starting broiler chicks. Poult. Sci..

[126] Grimble R.F. (2001). Nutritional modulation of immune function. Proc. Nutr. Soc..

[127] Berger M.M., Chiolero R. (2007). Antioxidant supplementation in sepsis and systemic inflammatory response syndrome. Crit. Care Med..

[128] Wischmeyer P.E., Lynch J.P., Liedel J., Wolfson R.L., Riehm J., Gottlieb L.J., Kahana M. (2001). Glutamine administration reduces Gram-negative bacteremia in severely burned patients: A prospective, randomized, double-blind trial versus isonitrogenous control. Crit. Care Med..

[129] Wu D., Su S., Zha X., Yan W., Yang G., Huang Q., Yang Y., Lin X., Fan S., Peng X. (2022). Glutamine promotes O-GlcNAcylation of G6PD and inhibits AGR2 S-glutathionylation to maintain the intestinal mucus barrier in burned septic mice. Redox Biol..

[130] Rodriguez P.C., Ochoa A.C., Al-Khami A.A. (2017). Arginine metabolism in myeloid cells shapes innate and adaptive immunity. Front. Immunol..

[131] Dong Y., Jiang W.-D., Wu P., Liu Y., Kuang S.-Y., Tang L., Tang W.-N., Zhou X.-Q., Feng L. (2022). Nutritional digestion and absorption, metabolism fates alteration was associated with intestinal function improvement by dietary threonine in juvenile grass carp (*Ctenopharyngodon Idella*). Aquaculture.

[132] Wróblewska J., Złocińska H., Wróblewski M., Nuszkiewicz J., Woźniak A. (2025). The role of vitamins in pediatric urinary tract infection: Mechanisms and integrative strategies. Biomolecules.

[133] Min Y., Niu Z., Sun T., Wang Z., Jiao P., Zi B., Chen P., Tian D., Liu F. (2018). Vitamin E and vitamin C supplementation improves antioxidant status and immune function in oxidative-stressed breeder roosters by up-regulating expression of GSH-Px gene. Poult. Sci..

[134] Arthur J.R., McKenzie R.C., Beckett G.J. (2003). Selenium in the immune system. J. Nutr..

[135] Wen C., Yan W., Zheng J., Ji C., Zhang D., Sun C., Yang N. (2018). Feed efficiency measures and their relationships with production and meat quality traits in slower growing broilers. Poult. Sci..

[136] Sugiharto S., Handayani F.R., Adli D.N., Sholikin M.M., Ujilestari T. (2026). Enzyme-assisted valorization of agro-industrial byproducts for sustainable and efficient broiler production. Vet. World.

[137] Varkoohi S., Shahr Babak M.M., Pakdel A., Javaremi A.N., Zaghari M., Kause A. (2010). Response to selection for feed conversion ratio in Japanese quail. Poult. Sci..

[138] Badshah F., Latif A., Maqbool M., Ahmad M., Zafar M.B., Ali M.A., Faiz S., Sarwar M., Adnan M., Sohail M. (2023). Effect of enzyme supplementation on the performance of broiler chickens. Biol. Clin. Sci. Res. J..

[139] Opazo R., Salinas C., Villasante A. (2025). Formulation strategies of probiotics in broilers: Systematic review and meta-analysis of their effects on production performance. Front. Anim. Sci..

[140] El-Fateh M., Bilal M., Zhao X. (2024). Effect of antibiotic growth promoters (AGPs) on feed conversion ratio (FCR) of broiler chickens: A meta-analysis. Poult. Sci..

[141] Pagthinathan M., Inthujaa S., Wijekoon W. (2019). Effect of litter materials on broiler performance. Sch. J. Agric. Vet. Sci..

[142] Rauf U., Batool F., Usman M., Mohsan M., Assad M.A., Ali M.M., Mujtaba M., Awan G.M., Iftikhar M.A., Abdullah S.S. (2024). Effect of various types of housing systems in poultry especially broilers. Indus J. Biosci. Res..

[143] Gickel J., Hankel J., Abd El-Wahab A., Arnold C.P., Visscher C. (2025). The effect of pathogen-induced diseases on the carbon footprint of broiler chickens. Poult. Sci..

[144] Yang L., Wang X., He T., Xiong F., Chen X., Chen X., Jin S., Geng Z. (2020). Association of residual feed intake with growth performance, carcass traits, meat quality, and blood variables in native chickens. J. Anim. Sci..

[145] Ramankevich A., Danko S., Banaszkiewicz R., Kasperek K., Zięba G. (2025). Residual feed intake as a behavioral, nutritional and economic criterion in poultry production. Animals.

[146] Pirzado S.A., Zheng A., Chen J., Zou Z., Liu G. (2026). Comparative evaluation of phytate and phytase addition on growth performance and nutrient utilization efficiency in broilers and ducks. Poult. Sci..

[147] Chen C., Su Z., Li Y., Luan P., Wang S., Zhang H., Xiao F., Guo H., Cao Z., Li H. (2020). Estimation of the genetic parameters of traits relevant to feed efficiency: Result from broiler lines divergent for high or low abdominal fat content. Poult. Sci..

[148] Friggens N.C., Brun-Lafleur L., Faverdin P., Sauvant D., Martin O. (2011). Advances in predicting nutrient partitioning in the dairy cow: Recognizing the central role of genotype and its expression through time. animal.

[149] Chrystal P.V., Moss A.F., Khoddami A., Naranjo V., Selle P.H., Liu S.Y. (2019). Impacts of reduced-crude protein diets on key parameters in male broiler chickens offered maize-based diets. Poult. Sci..

[150] Zeng X., Javid A., Fraley G.S., Tian G., Zhang K., Bai S., Ding X., Wang J., Liu Y., Xuan Y. (2025). Evaluation of alterations in nutrient utilization and intestinal health in response to heat stress in Pekin ducks based on a pair-feeding experimental design. Animals.

[151] Cambra-López M., Marín-García P.J., Lledó C., Cerisuelo A., Pascual J.J. (2022). Biomarkers and De Novo protein design can improve precise amino acid nutrition in broilers. Animals.

[152] Musigwa S., Cozannet P., Asiamah C.A., Wu S.-B. (2024). Effects of dietary protein levels, net energy levels, and essential amino acid-to-true protein ratios on broiler performance. Animals.

[153] Chrystal P.V., Moss A.F., Khoddami A., Naranjo V., Selle P.H., Liu S.Y. (2019). Effects of reduced crude protein levels, dietary electrolyte balance, and energy density on the performance of broiler chickens offered maize-based diets with evaluations of starch, protein, and amino acid metabolism. Poult. Sci..

[154] Cho I., An S.H., Yoon J.H., Namgung N., Kong C. (2023). Growth performance and nitrogen excretion of broiler chickens fed low protein diets supplemented with crystalline amino acids. J. Anim. Sci. Technol..

[155] Jiang D., Zhang J., Ji Y., Dai Z., Yang Y., Wu Z. (2025). Glutamate supplementation regulates nitrogen metabolism in the colon and liver of weaned rats fed a low-protein diet. Nutrients.

[156] Ospina-Rojas I.C., Murakami A.E., Eyng C., Nunes R.V., Duarte C.R.D.A., Diaz-Vargas M. (2012). Commercially available amino acid supplementation of low-protein diets for broiler chickens with different ratios of digestible glycine+serine:Lysine. Poult. Sci..

[157] Ospina-Rojas I.C., Murakami A.E., de Oliveira C.A.L., Guerra A.F.Q.G. (2013). Supplemental glycine and threonine effects on performance, intestinal mucosa development, and nutrient utilization of growing broiler chickens. Poult. Sci..

[158] Chrystal P.V., Moss A.F., Yin D., Khoddami A., Naranjo V.D., Selle P.H., Liu S.Y. (2020). Glycine equivalent and threonine inclusions in reduced-crude protein, maize-based diets impact on growth performance, fat deposition, starch-protein digestive dynamics and amino acid metabolism in broiler chickens. Anim. Feed Sci. Technol..

[159] Liu Y., Zhao Y., Ma J., Guo S., Gao X., Wang B., Gong L., Lv Z., Guo Y. (2024). Optimal glycine allowance levels in low-protein diets and the dynamic requirement model for broilers. Poult. Sci..

[160] Dean D.W., Bidner T.D., Southern L.L. (2006). Glycine supplementation to low protein, amino acid-supplemented diets supports optimal performance of broiler chicks. Poult. Sci..

[161] Napolitano G., Fasciolo G., Venditti P. (2021). Mitochondrial management of reactive oxygen species. Antioxidants.

[162] Greenhalgh S., Lemme A., de Paula Dorigam J.C., Chrystal P.V., Macelline S.P., Liu S.Y., Selle P.H. (2022). Dietary crude protein concentrations, feed grains, and whey protein interactively influence apparent digestibility coefficients of amino acids, protein, starch, and performance of broiler chickens. Poult. Sci..

[163] Macelline S.P., Wickramasuriya S.S., Cho H.M., Kim E., Shin T.K., Hong J.S., Kim J.C., Pluske J.R., Choi H.J., Hong Y.G. (2019). Broilers fed a low protein diet supplemented with synthetic amino acids maintained growth performance and retained intestinal integrity while reducing nitrogen excretion when raised under poor sanitary conditions. Poult. Sci..

[164] Alabi O.O., Shoyombo A.J., Akpor O.B., Oluba O.M., Adeyonu A.G. (2019). Exogenous enzymes and the digestibility of nutrients by broilers: A mini review. Int. J. Poult. Sci..

[165] Cowieson A.J., Acamovic T., Bedford M.R. (2006). Phytic acid and phytase: Implications for protein utilization by poultry. Poult. Sci..

[166] Shanmugam G. (2018). Characteristics of phytase enzyme and its role in animal nutrition. Int. J. Curr. Microbiol. Appl. Sci..

[167] Selle P.H., Macelline S.P., Chrystal P.V., Liu S.Y. (2023). The contribution of phytate-degrading enzymes to chicken-meat production. Animals.

[168] Morgan N.K., Bhuiyan M., Hopcroft R. (2022). Non-starch polysaccharide degradation in the gastrointestinal tract of broiler chickens fed commercial-type diets supplemented with either a single dose of xylanase, a double dose of xylanase, or a cocktail of non-starch polysaccharide-degrading enzymes. Poult. Sci..

[169] Madigan-Stretton J., Mikkelsen D., Assadi Soumeh E. (2020). Multienzyme super-dosing in broiler chicken diets: The implications for gut morphology, microbial profile, nutrient digestibility, and bone mineralization. Animals.

[170] Dao H.T., Moss A.F. (2025). Canola meal: A sustainable protein source for poultry diets. Animals.

[171] Zangoli C., Chrysanthopoulos S., Pocheville S., Fernandes E., Zappaterra M., de Almeida A.M., Fangueiro D. (2026). Using Spirulina (Limnospira platensis) as an alternative feedstuff for poultry: Effects on ammonia and greenhouse gas emissions from excreta during storage. Poult. Sci..

[172] Ling S.D., Shafiee M., Longworth Z., Vatanparast H., Tabatabaei M., Jung L.H. (2024). Black soldier fly larvae meal (BSFLM) as an alternative protein source in sustainable aquaculture production: A scoping review of its comprehensive impact on shrimp and prawn farming. Anim. Feed Sci. Technol..

[173] Wu X., Wang M., Luo S., Zhou Z., Wang Y., Du G., Chen J., Liu X. (2025). Dual enhancement of mycoprotein nutrition and sustainability via CRISPR-mediated metabolic engineering of *Fusarium venenatum*. Trends Biotechnol..

[174] Dickson R., Mansouri S.S. (2025). Sustainable production of fermentation-based novel proteins. Comput. Chem. Eng..

[175] Chethan M.N., Chandrashekhar S., Mk V., Kumar M.A. (2026). Nutritional composition and potential of silkworm pupal meal as an alternative protein source in animal feed. Int. J. Agric. Food Sci..

[176] Arshad M., Abbas G., Jaffery S., Hashmi A.H., Ussain I., Mustafa A., Rehman A., Iqbal A., Khan R., Mahmood A. (2023). Earthworm meal: A novel non-conventional feed ingredient for sustainable poultry production. Pak. J. Sci..

[177] Sońta M., Rekiel A., Więcek J., Batorska M., Puppel K. (2021). Alternative protein sources vs. GM soybean meal as feedstuff for pigs—Meat quality and health-promoting indicators. Animals.

[178] El-Deek A., Abdel-Wareth A., Osman M., El-Shafey M., Khalifah A., Elkomy A., Lohakare J. (2020). Alternative feed ingredients in the finisher diets for sustainable broiler production. Sci. Rep..

[179] Boskovic Cabrol M., Martins J.C., Malhão L.P., Alves S.P., Bessa R.J.B., Almeida A.M., Raymundo A., Lordelo M. (2022). Partial replacement of soybean meal with *Chlorella vulgaris* in broiler diets influences performance and improves breast meat quality and fatty acid composition. Poult. Sci..

[180] Bortolini D.G., Maciel G.M., Fernandes I.d.A.A., Pedro A.C., Rubio F.T.V., Branco I.G., Haminiuk C.W.I. (2022). Functional properties of bioactive compounds from *Spirulina* spp.: Current status and future trends. Food Chem. Mol. Sci..

[181] Lobo R.R., Siregar M.U., da Silva S.S., Monteiro A.R., Salas-Solis G., Vicente A.C.S., Vinyard J.R., Johnson M.L., Ma S., Sarmikasoglou E. (2024). Partial replacement of soybean meal with microalgae biomass on in vitro ruminal fermentation. J. Dairy Sci..

[182] Bernal-Cabas M., Kumar K., Terpstra O., Bogaard S.v.D., Ammar A.B., Mäkinen S., Herwig L., de Almeida M., Tervasmäki P., Blank L.M. (2026). Food production from air: Gas precision fermentation with hydrogen-oxidising bacteria. Trends Biotechnol..

[183] Gopinger E., Xavier E.G., Elias M.C., Catalan A.A.S., Castro M.L.S., Nunes A.P., Roll V.F.B. (2014). The effect of different dietary levels of canola meal on growth performance, nutrient digestibility, and gut morphology of broiler chickens. Poult. Sci..

[184] Horodincu L., Proca A.C., Șlencu B.G., Trifan A., Pavel G., Solcan G., Solcan C. (2025). Modulating effects of grape pomace on the intestinal antioxidative and inflammatory status in fattening pigs. Agriculture.

[185] Dal Bosco A., Mourvaki E., Cardinali R., Servili M., Sebastiani B., Ruggeri S., Mattioli S., Taticchi A., Esposto S., Castellini C. (2012). Effect of dietary supplementation with olive pomaces on the performance and meat quality of growing rabbits. Meat Sci..

[186] Tie Y., Li L., Liu J., Liu C., Fu J., Xiao X., Wang G., Wang J. (2020). Two-step biological approach for treatment of rapeseed meal. J. Food Sci..

[187] Moss A.F., Chrystal P.V., Cadogan D.J., Wilkinson S., Crowley T.M., Choct M. (2021). Precision feeding and precision nutrition: A paradigm shift in broiler feed formulation?. Anim. Biosci..

[188] Sharifuzzaman M., Mun H.-S., Lagua E.B., Hasan M.K., Kang J.-G., Kim Y.-H., Mehtab A., Park H.-R., Yang C.-J. (2026). Advances in audio-based artificial intelligence for respiratory health and welfare monitoring in broiler chickens. AI.

[189] You J., van der Klein S., Lou E., Zuidhof M.J. (2020). Application of random forest classification to predict daily oviposition events in broiler breeders fed by precision feeding system. Comput. Electron. Agric..

[190] Zuidhof M.J., Fedorak M.V., Ouellette C.A., Wenger I.I. (2017). Precision feeding: Innovative management of broiler breeder feed intake and flock uniformity. Poult. Sci..

[191] Aydın A., Bahr C., Berckmans D. (2015). A real-time monitoring tool to automatically measure the feed intakes of multiple broiler chickens by sound analysis. Comput. Electron. Agric..

[192] Aerts J.-M., Van Buggenhout S., Vranken E., Lippens M., Buyse J., Decuypere E., Berckmans D. (2003). Active control of the growth trajectory of broiler chickens based on online animal responses. Poult. Sci..

[193] Selionova M.I., Trukhachev V.I., Zagarin A.Y., Kulikov E.I., Dmitrenko D.M., Martynova V.N., Kravchenko A.K., Vertiprakhov V.G. (2024). Expression of genes related to meat productivity, metabolic and morphological significance of broiler chickens with the use of nutritional phytochemicals. Animals.

[194] Chatterjee A. (2026). The future of aquaculture: Integrating artificial intelligence and machine learning for sustainable productivity. Biotechnol. Acta.

[195] Prudhomme R., O’Donoghue C., Ryan M., Styles D. (2021). Defining national biogenic methane targets: Implications for national food production & climate neutrality objectives. J. Environ. Manag..

[196] Chadwick D., Sommer S., Thorman R., Fangueiro D., Cardenas L., Amon B., Misselbrook T. (2011). Manure management: Implications for greenhouse gas emissions. Anim. Feed Sci. Technol..

[197] Petersen S.O., Blanchard M., Chadwick D.R., del Prado A., Edouard N., Mosquera J., Sommer S.G. (2013). Manure management for greenhouse gas mitigation. animal.

[198] Alves E.C., Alves I.H.S., Soares B.B., Borges A.F., Jalal A., Jani A.D., Abreu-Junior C.H., Capra G.F., Nogueira T.A.R. (2023). Resource recovery of biological residues from the Brazilian poultry industry in mitigating environmental impacts: A life cycle assessment (LCA) approach. J. Clean. Prod..

[199] Leinonen I., Williams A., Wiseman J., Guy J.H., Kyriazakis I. (2011). Predicting the environmental impacts of chicken systems in the United Kingdom through a life cycle assessment: Egg production systems. Poult. Sci..

[200] Linton N., Machado P., Deen B., Wagner-Riddle C., Dunfield K. (2020). Long-term diverse rotation alters nitrogen cycling bacterial groups and nitrous oxide emissions after nitrogen fertilization. Soil Biol. Biochem..

[201] Spawn S., Lark T.J., Gibbs H. (2019). Carbon emissions from cropland expansion in the United States. Environ. Res. Lett..

[202] Bevilacqua M., Ciarapica F.E., Giacchetta G., Marchetti B. (2010). A carbon footprint analysis in the textile supply chain. Int. J. Sustain. Eng..

[203] de Vries M., de Boer I.J.M. (2009). Comparing environmental impacts for livestock products: A review of life cycle assessments. Livest. Sci..

[204] Tetteh H., Bala A., Fullana-i-Palmer P., Balcells M., Margallo M., Aldaco R., Puig R. (2022). Carbon footprint: The case of four chicken meat products sold on the Spanish market. Foods.

[205] Chen L., Zhang R., Li Y., Zhang Y., Fang W., Zhang P., Zhang G. (2023). Internal driving mechanisms of microbial community and metabolism for granular activated carbon enhancing anaerobic digestion of high-strength sulfate organic wastewater. Chem. Eng. J..

[206] Saiz E., Sgouridis F., Drijfhout F.P., Peichl M., Nilsson M.B., Ullah S. (2021). Chronic atmospheric reactive nitrogen deposition suppresses biological nitrogen fixation in peatlands. Environ. Sci. Technol..

[207] Hernández F., López M.J., Martínez-Miró S., Megías M.D., Catalá P., Madrid J. (2012). Effect of low-protein diets and single sex on production performance, plasma metabolites, digestibility, and nitrogen excretion in 1- to 48-day-old broilers. Poult. Sci..

[208] Nanto-Hara F., Ema T., Ohtsu H. (2026). Amino-acid-balanced low-protein diets reduce nitrogen excretion without affecting growth performance in broilers. Animals.

[209] Wang Y., Li X., Yang J., Tian Z., Sun Q., Xue W., Dong H. (2018). Mitigating greenhouse gas and ammonia emissions from beef cattle feedlot production: A system meta-analysis. Environ. Sci. Technol..

[210] Chen X., Wen Y., Li Z., Jin W., Lin H., Liu J. (2025). Influence of *Bacillus subtilis* supplementation on growth performance and gut microbial composition in broiler chickens based on low-protein diets. Poult. Sci..

[211] Strifler P., Horváth B., Such N., Dublecz K., Pál L. (2024). Effects of different dietary threonine and glycine supplies in broilers fed low-protein diets. Front. Vet. Sci..

[212] Hilliar M., Hargreave G., Girish C.K., Barekatain R., Wu S.-B., Swick R.A. (2020). Using crystalline amino acids to supplement broiler chicken requirements in reduced protein diets. Poult. Sci..

[213] Henders S., Persson U.M., Kästner T. (2015). Trading forests: Land-use change and carbon emissions embodied in production and exports of forest-risk commodities. Environ. Res. Lett..

[214] Noojipady P., Morton C., Macedo M.N., Victoria C.D., Huang C., Gibbs K.H., Bolfe É.L. (2017). Forest carbon emissions from cropland expansion in the Brazilian Cerrado biome. Environ. Res. Lett..

[215] Leite C.C., Costa M.H., Soares-Filho B., Hissa L.D.B.V. (2012). Historical land use change and associated carbon emissions in Brazil from 1940 to 1995. Glob. Biogeochem. Cycles.

[216] Baumann M., Gasparri I., Piquer-Rodríguez M., Gavier Pizarro G., Griffiths P., Hostert P., Kuemmerle T. (2016). Carbon emissions from agricultural expansion and intensification in the Chaco. Glob. Change Biol..

[217] Morton D.C., DeFries R.S., Shimabukuro Y.E., Anderson L.O., Arai E., del Bon Espirito-Santo F., Freitas R., Morisette J. (2006). Cropland expansion changes deforestation dynamics in the southern Brazilian Amazon. Proc. Natl. Acad. Sci. USA.

[218] Gollnow F., de Barros Viana Hissa L., Rufin P., Lakes T. (2018). Property-level direct and indirect deforestation for soybean production in the Amazon region of Mato Grosso, Brazil. Land Use Policy.

[219] DeFries R.S., Morton D.C., van der Werf G.R., Giglio L., Collatz G.J., Randerson J.T., Houghton R.A., Kasibhatla P.K., Shimabukuro Y. (2008). Fire-related carbon emissions from land use transitions in southern Amazonia. Geophys. Res. Lett..

[220] Yoon J.H., Lourenço J.M., Olukosi O.A. (2026). Interactive effects of dietary crude protein reduction and resistant starch inclusion on growth performance and cecal microbial fermentation in broiler chickens. Poult. Sci..

[221] Zhou Y., Dong H., Xin H., Zhu Z., Huang W., Wang Y. (2018). Carbon footprint assessment of a large-scale pig production system in Northern China: A case study. Trans. ASABE.

[222] Hechelmann R.H., Paris A., Buchenau N., Ebersold F. (2023). Decarbonisation strategies for manufacturing: A technical and economic comparison. Renew. Sustain. Energy Rev..

[223] Rissman J.R., Bataille C., Masanet E., Aden N., Morrow W.R., Zhou N., Elliott N., Dell R., Heeren N., Huckestein B. (2020). Technologies and policies to decarbonize global industry: Review and assessment of mitigation drivers through 2070. Appl. Energy.

[224] Navales R.A.S., Tokach M.D., DeRouchey J.M., Gaffield K.N., Woodworth J.C., Goodband R.D., Gebhardt J.T., Euken R.M., Dekkers J.C.M. (2025). Technologies and practices to improve feed and nutrient utilization by pigs. J. Anim. Sci..

[225] Wang Y.-L., Wang W., Wu Q., Yang H. (2022). The release and catabolism of ferulic acid in plant cell wall by rumen microbes: A review. Anim. Nutr..

[226] Etxeberría U., Arias N., Boqué N., Macarulla M.T., Portillo M.P., Martínez J.A., Milagro F.I. (2015). Reshaping faecal gut microbiota composition by the intake of trans-resveratrol and quercetin in high-fat sucrose diet-fed rats. J. Nutr. Biochem..

[227] Adeoye A.A., Yomla R., Jaramillo-Torres A., Rodiles A., Merrifield D.L., Davies S.J. (2016). Combined effects of exogenous enzymes and probiotic on Nile tilapia (Oreochromis niloticus) growth, intestinal morphology and microbiome. Aquaculture.

[228] Wealleans A., Walsh M.C., Romero L.F., Ravindran V. (2017). Comparative effects of two multi-enzyme combinations and a Bacillus probiotic on growth performance, digestibility of energy and nutrients, disappearance of non-starch polysaccharides, and gut microflora in broiler chickens. Poult. Sci..

[229] Zheng J., Geng J., Li J., Li D., Yang S., Lu X., Liu J., Zhang C., Sun H. (2025). Harnessing microalgae for protein production: Advances, functional properties, and industrial potential. Future Foods.

[230] Taelman S.E., De Meester S., van Dijk W., da Silva V., Dewulf J. (2015). Environmental sustainability analysis of a protein-rich livestock feed ingredient in The Netherlands: Microalgae production versus soybean import. Resour. Conserv. Recycl..

[231] Shanmugam K., Bryngelsson S., Östergren K., Hallström E. (2023). Climate impact of plant-based meat analogues: A review of life cycle assessments. Sustain. Prod. Consum..

[232] Petrychenko V., Korniichuk O. (2022). Scientific provision of feed production in marital state conditions. Feed. Feed Prod..

[233] Brás T., Guerra V., Torrado I., Lourenço P., Carvalheiro F., Duarte L.C., Neves L.A. (2013). Detoxification of hemicellulosic hydrolysates from extracted olive pomace by diananofiltration. Process Biochem..

[234] Cheng H., Liu X., Xiao Q., Zhang F., Liu N., Tang L., Wang J., Ma X., Tan B., Chen J. (2022). Rapeseed meal and its application in pig diet: A review. Agriculture.

[235] Hou Y., Velthof G.L., Oenema O. (2014). Mitigation of ammonia, nitrous oxide and methane emissions from manure management chains: A meta-analysis and integrated assessment. Glob. Change Biol..

[236] Aguerre M.J., Wattiaux M.A., Powell J.M., Broderick G.A., Arndt C. (2011). Effect of forage-to-concentrate ratio in dairy cow diets on emission of methane, carbon dioxide, and ammonia, lactation performance, and manure excretion. J. Dairy Sci..

[237] Morey L., Bach À., Sabrià D., Riau V., Fernández B., Terré M. (2023). Effectiveness of precision feeding in reducing N excretion in dairy cattle. Anim. Feed Sci. Technol..

[238] Shen X., Jin H., Zhao F., Kwok L.-Y., Zhao Z., Sun Z. (2024). Short-term probiotic supplementation affects the diversity, genetics, growth, and interactions of the native gut microbiome. iMeta.

[239] Cayuela M.L., Jeffery S., Van Zwieten L. (2015). The molar H: Corg ratio of biochar is a key factor in mitigating N_2_O emissions from soil. Agric. Ecosyst. Environ..

[240] Li D., Watson C.J., Yan M.J., Lalor S., Rafique R., Hyde B., Lanigan G., Richards K.G., Holden N.M., Humphreys J. (2013). A review of nitrous oxide mitigation by farm nitrogen management in temperate grassland-based agriculture. J. Environ. Manag..

[241] Sell-Kubiak E., Wimmers K., Reyer H., Szwaczkowski T. (2017). Genetic aspects of feed efficiency and reduction of environmental footprint in broilers: A review. J. Appl. Genet..

[242] Zuidhof M.J. (2018). Lifetime productivity of conventionally and precision-fed broiler breeders. Poult. Sci..

[243] Matthews H.S., Hendrickson C., Weber C.L. (2008). The importance of carbon footprint estimation boundaries. Environ. Sci. Technol..

[244] Sterling K.G., Pesti G.M., Bakalli R.I. (2006). Performance of different broiler genotypes fed diets with varying levels of dietary crude protein and lysine. Poult. Sci..

[245] Wen C., Jiang X., Ding L., Wang T., Zhou Y. (2016). Effects of dietary methionine on growth performance, meat quality and oxidative status of breast muscle in fast- and slow-growing broilers. Poult. Sci..

[246] Olayiwola S.F., Adedokun S.A. (2025). The efficacy of feed additives in alleviating heat stress and supporting gut health in poultry. Front. Anim. Sci..

[247] Kumar A., Toghyani M., Kheravii S.K., Pineda L., Han Y., Swick R.A., Wu S.B. (2021). Organic acid blends improve intestinal integrity, modulate short-chain fatty acids profiles and alter microbiota of broilers under necrotic enteritis challenge. Anim. Nutr..

[248] VandeHaar M.J., St-Pierre N.R. (2006). Major advances in nutrition: Relevance to the sustainability of the dairy industry. J. Dairy Sci..

[249] Petersen S.O., Sommer S.G. (2011). Ammonia and nitrous oxide interactions: Roles of manure organic matter management. Anim. Feed Sci. Technol..

[250] Zhu W., Winter M.G., Byndloss M.X., Spiga L., Duerkop B.A., Hughes E.R., Büttner L., Romão E.L., Behrendt C.L., Lopez C.A. (2018). Precision editing of the gut microbiota ameliorates colitis. Nature.

[251] Hadinia S., Carneiro P.R.O., Ouellette C.A., Zuidhof M.J. (2018). Energy partitioning by broiler breeder pullets in skip-a-day and precision feeding systems. Poult. Sci..

[252] Belloir P., Méda B., Lambert W., Corrent E., Juin H., Lessire M., Tesseraud S. (2017). Reducing the CP content in broiler feeds: Impact on animal performance, meat quality and nitrogen utilization. animal.

[253] Wang L., Bai X., Yan T. (2017). Greenhouse gas emissions from pig and poultry production sectors in China from 1960 to 2010. J. Integr. Agric..

[254] Elahi U., Wang J., Ma Y., Wu S., Qi G., Zhang H. (2020). The response of broiler chickens to dietary soybean meal reduction with glycine and cysteine inclusion at marginal sulfur amino acids (SAA) deficiency. Animals.

[255] Sampath V., Pineda L., Hambrecht E., Kim I.H. (2025). Synergistic blend of natural essential oils improved growth performance and gut barrier integrity in broilers by alleviating intestinal inflammation. Poult. Sci..

[256] Wang M., Zhang Y., Zhu C., Yao X., Zheng Z., Tian Z., Cai X. (2021). EkFLS overexpression promotes flavonoid accumulation and abiotic stress tolerance in plant. Physiol. Plant..

[257] Gratta F., Birolo M., Sacchetto R., Radaelli G., Xiccato G., Ballarin C., Bertotto D., Piccirillo A., Petracci M., Maertens L. (2019). Effect of feed restriction timing on live performance, breast myopathy occurrence, and muscle fiber degeneration in 2 broiler chicken genetic lines. Poult. Sci..

[258] Woyengo T.A., Bach Knudsen K.E., Børsting C.F. (2023). Low-protein diets for broilers: Current knowledge and potential strategies to improve performance and health, and to reduce environmental impact. Anim. Feed Sci. Technol..

[259] Kamran Z., Sarwar M., Nisa M., Nadeem M.A., Ahmad S., Mushtaq T., Ahmad T., Shahzad M.A. (2008). Effect of lowering dietary protein with constant energy to protein ratio on growth, body composition and nutrient utilization of broiler chicks. Asian-Australas. J. Anim. Sci..

[260] Jiang W., Li G., Xu J., Qin Y., Wang L., Wang H. (2025). Precision feeding systems in animal husbandry: Guiding rabbit farming from concept to implementation. Agriculture.

[261] Gavalas N.P., Kalburtji K.L., Kokkini S., Mamolos A.P., Veresoglou D.S. (2011). Ecotypic variation in plant characteristics for Origanum vulgare subsp. hirtum populations. Biochem. Syst. Ecol..

[262] Zengin G., Cvetanović A., Gašić U., Dragićević M., Stupar A., Uysal A., Şenkardes I., Sinan K.I., Picot-Allain M.C.N., Ak G. (2020). UHPLC-LTQ OrbiTrap MS analysis and biological properties of Origanum vulgare subsp. viridulum obtained by different extraction methods. Ind. Crops Prod..

[263] Cai W., Jusoh S., Yue X. (2026). Digitalization and sustainable industrial low-carbon transformation: Synergistic effects, policy tools, technical pathways, and financial innovation. Sustainability.

[264] (2006). Environmental Management—Life Cycle Assessment—Principles and Framework.

[265] (2006). Environmental Management—Life Cycle Assessment—Requirements and Guidelines.

[266] European Commission (2021). Commission Recommendation of 16 December 2021 on the Use of the Environmental Footprint Methods to Measure and Communicate the Life Cycle Environmental Performance of Products and Organisations, C (2021) 9332 Final.

